# Cyclodextrin Interactions with Water-Soluble Vitamins: Biological Relevance, Stability, Delivery, and Translational Implications—A Critical Review

**DOI:** 10.3390/biology15181637

**Published:** 2026-09-16

**Authors:** Zuzanna Stolarek, Marcin Gackowski, Łukasz Szeleszczuk

**Affiliations:** 1 Department of Organic and Physical Chemistry, Medical University of Warsaw, 1 Banacha Str., 02-097 Warsaw, Poland; s092628@student.wum.edu.pl (Z.S.); lukasz.szeleszczuk@wum.edu.pl (Ł.S.); 2 Department of Toxicology and Bromatology, Faculty of Pharmacy, L. Rydygier Collegium Medicum in Bydgoszcz, Nicolaus Copernicus University in Torun, 2 Jurasza Str., 85-089 Bydgoszcz, Poland

**Keywords:** cyclodextrins, water-soluble vitamins, B vitamins, vitamin C, vitamin stability, drug delivery, bioavailability, biomolecular interactions

## Abstract

Water-soluble vitamins participate in essential metabolic, redox, and coenzyme-dependent processes, yet their effective delivery can be limited by chemical instability, degradation during processing or storage, restricted membrane transport, and formulation-dependent loss of biological availability. Cyclodextrins have been widely investigated as molecular hosts and functional components of delivery systems capable of modifying these properties. This critical narrative review evaluates cyclodextrin systems involving thiamine, riboflavin, nicotinic acid and nicotinamide, pantothenate, vitamin B6 vitamers, biotin, folates, cobalamins, and vitamin C, with particular emphasis on the relationship between molecular interaction mechanisms and functional or biological outcomes. The available evidence demonstrates that cyclodextrins can protect selected vitamins against photochemical, thermal, and chemical degradation, modify release profiles, facilitate local or transdermal delivery, and support pharmaceutical, food, biomedical, and analytical applications. However, these effects cannot always be attributed to classical cavity inclusion. Depending on vitamin structure, hydration, charge, and molecular size, the underlying mechanism may involve partial inclusion, external hydrogen bonding, ion pairing, multivalent association, or physical entrapment within cyclodextrin-containing materials. Distinguishing these mechanisms is essential for interpreting whether an observed improvement in stability, permeability, or biological performance can reasonably be linked to molecular complexation. Importantly, evidence that cyclodextrin association increases systemic vitamin bioavailability in vivo remains limited, and improvements in solubility or release should not automatically be considered evidence of enhanced biological availability. Future studies should therefore integrate rigorous molecular characterization with permeability, cellular, pharmacokinetic, and in vivo evaluation. Such an approach can establish a mechanistic link between cyclodextrin–vitamin interactions and their biological and translational significance.

## Simple Summary

Water-soluble vitamins are essential regulators and cofactors of numerous biological processes, but their biological effectiveness can be limited by chemical degradation, formulation instability, restricted membrane transport, or insufficient retention at the site of administration. Cyclodextrins are widely used in pharmaceutical, food, and biomedical systems and can interact with vitamins through several molecular mechanisms. This review critically examines cyclodextrin systems involving vitamins B1, B2, B3, B5, B6, B7, B9, B12, and vitamin C, with particular emphasis on how the nature of these interactions affects vitamin stability, release, delivery, permeability, and biological performance. We distinguish true cavity inclusion from partial inclusion, external association, physical entrapment, and vitamin-functionalized cyclodextrin systems. This distinction is biologically relevant because different interaction mechanisms can lead to different effects on vitamin protection, availability, and delivery. Although cyclodextrin-based systems frequently improve stability or modify release, convincing evidence that these effects translate into increased in vivo bioavailability remains limited. Connecting molecular interaction mechanisms with biological and translational outcomes is therefore a key priority for future studies.

## 1. Introduction

Water-soluble vitamins comprise vitamin C and the chemically diverse B-group vitamins, which function mainly as coenzymes, coenzyme precursors, redox mediators, and regulators of essential metabolic pathways. Their classification is nutritional rather than structural: the group ranges from small heteroaromatic compounds to phosphorylated cofactors, folates, and the large corrinoid framework of cobalamins. Although aqueous solubility generally facilitates incorporation into liquid and semisolid products, it does not eliminate factors that can limit biological availability. Several water-soluble vitamins are sensitive to light, oxygen, temperature, moisture, pH, and interactions with other formulation components, and their stability varies substantially among vitamers and storage conditions [1,2].

For biologically active micronutrients, the amount initially incorporated into a formulation is not necessarily equivalent to the amount that ultimately remains chemically intact and available for absorption or local biological action. Degradation before administration, premature release, insufficient residence time, interactions with the surrounding matrix, and barriers to membrane transport may all reduce effective exposure. Moreover, high aqueous solubility does not guarantee high systemic availability: intestinal absorption can be transporter-limited, degradation may occur before uptake, and excess water-soluble vitamin can be rapidly eliminated. Consequently, delivery strategies for these vitamins should be evaluated according to the biological or functional limitation that they are intended to overcome, rather than according to solubility alone.

A variety of delivery systems, including electrospun fibers, spray-dried particles, hydrogels, liposomes, polymeric nanoparticles, and protein-based complexes, have therefore been investigated to protect water-soluble vitamins, control their release, improve local retention, and separate incompatible formulation components [3,4]. Cyclodextrins are particularly relevant in this context because they combine reversible molecular recognition with broad pharmaceutical and biomedical functionality. Native and substituted cyclodextrins can act as molecular hosts, formulation excipients, surface modifiers, crosslinking components, penetration-enhancing structures, and building blocks of supramolecular or polymeric delivery systems [5,6,7,8,9,10].

Understanding the molecular nature of vitamin–cyclodextrin association is important because different interaction mechanisms may produce different biological and formulation outcomes. Classical cavity inclusion can alter the local molecular environment of a vitamin and potentially protect labile structural regions, whereas shallow or external association may influence hydration, aggregation, or accessibility without complete encapsulation. In multicomponent carriers, cyclodextrins may instead modify membrane interactions, organize polymeric networks, or control release even when the vitamin itself does not occupy the cavity. Thus, evidence of improved stability, permeability, release, or cellular response should not automatically be interpreted as evidence of a classical inclusion complex.

This distinction is particularly important for water-soluble vitamins, whose strong hydration, ionization, and structural diversity make their cyclodextrin interactions substantially more complex than those of many poorly soluble hydrophobic guests. A vitamin and a cyclodextrin may form a true inclusion complex, a partial or external association, an aggregate, or may simply coexist within a multicomponent delivery system. Establishing which of these mechanisms operates is necessary not only for correct supramolecular description, but also for understanding the origin of the observed functional and biological effects.

Several recent reviews have addressed areas related to cyclodextrins, vitamin formulation, or micronutrient delivery, but their objectives differ substantially from those of the present work (Table 1). Reviews of cyclodextrin-based drug-delivery systems have primarily considered pharmaceutical active compounds in general, advanced cyclodextrin derivatives, nanocarriers, permeation enhancement, clinical development, and commercial products, without focusing specifically on water-soluble vitamins [6,7,11]. Other reviews have examined water-soluble vitamin delivery using technologies such as electrospinning and spray drying, but without specifically evaluating cyclodextrin–vitamin molecular interactions [1]. More recently, cyclodextrins have been reviewed as carriers for a broad range of micronutrients, including vitamins, iron, and iodine, with emphasis on formulation, stability, controlled release, and food, pharmaceutical, and cosmeceutical applications [12]. Encapsulation strategies for water-soluble vitamins have also been reviewed from a food-fortification perspective, with particular attention to folate and vitamin C, gastrointestinal stability, absorption, and metabolism [13]. In parallel, cyclodextrin complexes of the fat-soluble vitamins A, D, E, and K have been comprehensively reviewed with emphasis on their preparation, physicochemical characterization, and applications [14].

The present review addresses a complementary but distinct question. Rather than considering cyclodextrins primarily as formulation excipients or assuming that the presence of a vitamin and a cyclodextrin implies formation of an inclusion complex, we critically evaluate the molecular nature of the interaction and then examine whether the available evidence supports a functional or biological consequence. Thus, true cavity inclusion, partial inclusion, external association, physical entrapment, and covalent vitamin functionalization are considered separately, and the molecular evidence is related to chemical stability, release, permeability, cellular or biological effects, and translational evidence. This molecular-to-biological framework represents the principal distinction between the present review and earlier literature surveys.

**Figure 1:**
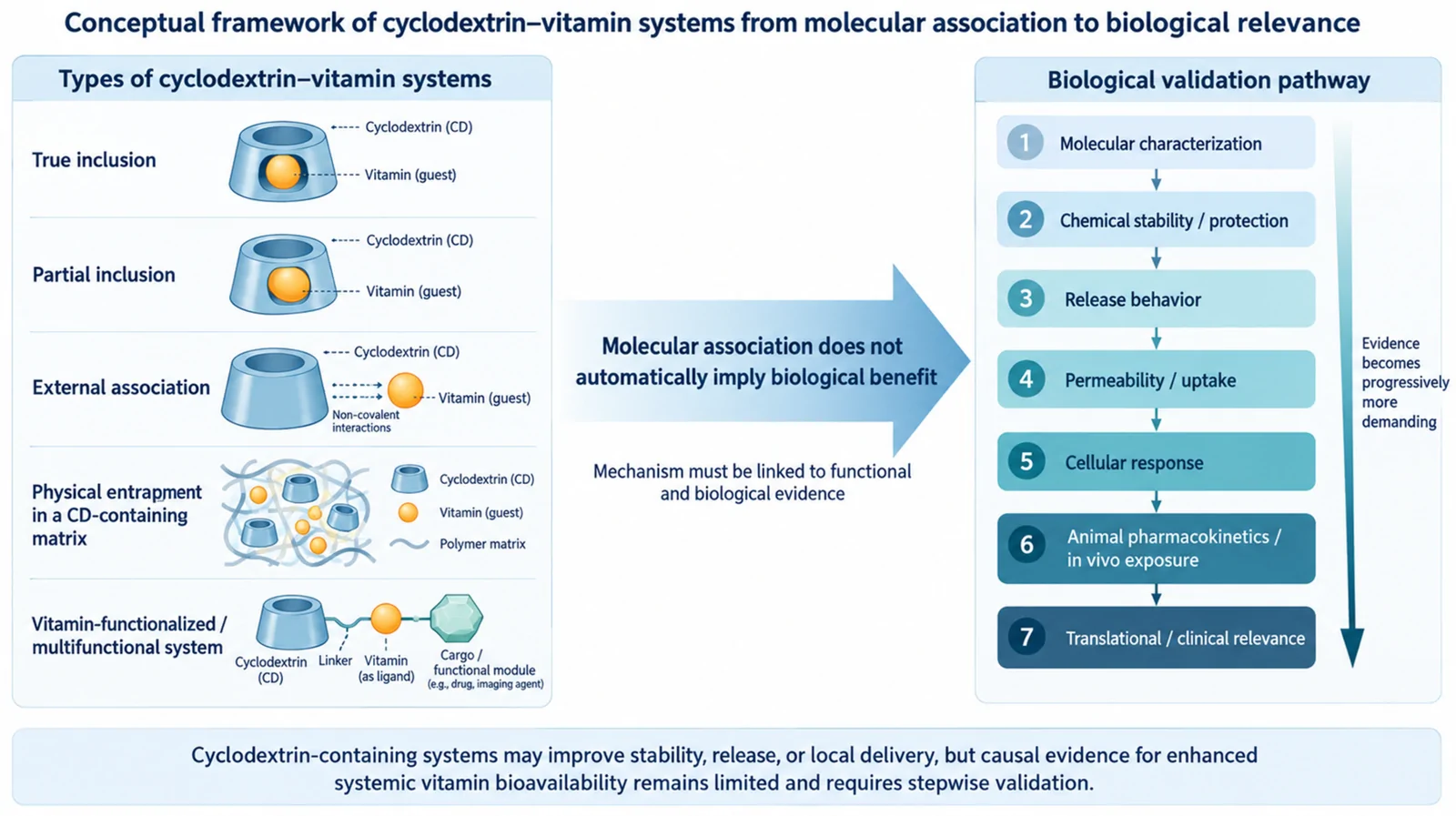
Conceptual framework of the present review. The figure distinguishes the principal modes of cyclodextrin–vitamin interaction—true inclusion, partial inclusion, external association, physical entrapment, and multifunctional or vitamin-functionalized systems—and relates them to successive levels of functional and biological validation. The scheme emphasizes that demonstration of molecular association does not automatically establish improved biological availability, which requires progressively stronger evidence from stability, release, permeability, cellular, pharmacokinetic, and translational studies.

Accordingly, the aim of the present review is to critically evaluate cyclodextrin–water-soluble vitamin systems from a molecular-to-biological perspective. We examine how vitamin structure, hydration, charge, and cyclodextrin type determine the mode and strength of association and, importantly, how these interactions relate to vitamin stability, release, permeability, delivery, and reported biological performance. Particular attention is given to distinguishing molecular evidence of complexation from formulation-level effects and to assessing whether reported improvements in physicochemical performance are supported by corresponding evidence of enhanced biological availability. By integrating molecular characterization with functional and translational outcomes, this review identifies the principal evidence gaps that currently prevent a direct connection between cyclodextrin complexation and improved in vivo vitamin performance. This conceptual framework is summarized schematically in Figure 1.

## 2. Literature Search and Study Selection

### 2.1. Literature Search

A targeted narrative literature search was conducted by Ł.S. and Z.S. using Scopus, Web of Science Core Collection, and Google Scholar. The search set was finalized in July 2026. Search terms combined “cyclodextrin” and the names of native or modified cyclodextrins with the common and chemical names of individual water-soluble vitamins and their principal vitamers, salts, and derivatives. Reference lists of relevant primary articles and reviews were also examined to identify earlier or otherwise difficult-to-retrieve studies.

Peer-reviewed original studies were prioritized when they directly addressed the preparation, characterization, association, formulation, stability, release, biological effects, or analytical use of a vitamin–cyclodextrin system. Studies of classical binary complexes were considered together with non-inclusion associations, cyclodextrin-containing delivery systems, functionalized cyclodextrins, and analytical platforms when these contributed to understanding the role of the host. Duplicate records were removed. Articles were excluded when the vitamin was only a quality marker, analytical standard, reducing reagent, or unrelated co-formulant, or when cyclodextrin complexed a different guest, unless the study provided a particularly relevant methodological or terminological example. Both authors assessed eligibility and resolved uncertain cases by discussion. Owing to substantial heterogeneity in host type, vitamin form, experimental conditions, and outcome measures, the evidence was synthesized qualitatively rather than by meta-analysis.

The search was additionally checked by backward citation screening of the included primary studies and relevant reviews. Searches were performed separately for each vitamin and major vitamer using both common and chemical names in combination with α-, β-, and γ-cyclodextrin and commonly used modified cyclodextrins. All relevant primary studies identified by this strategy and meeting the eligibility criteria described above were included in the qualitative synthesis.

The resulting evidence base was markedly uneven across vitamins. This imbalance reflects the available primary literature rather than differential selection of studies. Vitamin C and riboflavin have been investigated comparatively extensively, whereas only a small number of eligible studies were identified for pantothenate and for direct interactions involving free biotin or cobalamins. In the latter cases, much of the apparently related literature instead concerns covalently vitamin-functionalized cyclodextrins, multicomponent materials, or systems in which the vitamin is physically entrapped rather than acting as a molecular cyclodextrin guest. Such studies were retained only when they contributed directly to the mechanistic or biological distinctions considered in this review.

### 2.2. Evidence-Assessment Framework

To ensure consistent interpretation of the heterogeneous literature, the included molecularly characterized vitamin–cyclodextrin systems were reassessed using a three-grade evidence framework. The grading refers specifically to the strength of evidence for molecular association and cavity involvement and should not be interpreted as a measure of formulation efficacy or biological importance.

Grade A—strong molecular evidence. This grade requires quantitative solution-phase characterization of association together with cavity-sensitive or equivalent spatial evidence sufficient to establish the assigned interaction mode, including inclusion, exclusion, or predominantly external association. Typical qualifying evidence included reliable NMR or ITC determination of association and stoichiometry combined with intermolecular ROESY/NOESY correlations involving inward-facing cyclodextrin protons, or an equivalent orthogonal demonstration of guest penetration into the cavity. The experimental observations also had to be mutually consistent with the proposed interaction model.

Grade B—moderate molecular evidence. This grade was assigned when several complementary experimental techniques supported association, partial inclusion, or formation of a distinct host–guest system, but at least one key element required for Grade A was absent. Examples include systems lacking cavity-specific spatial evidence, independently established stoichiometry, or robust quantitative solution thermodynamics. One-dimensional NMR shifts, phase-solubility analysis, spectroscopic changes, calorimetric or solid-state characterization may contribute to Grade B when supported by complementary evidence but are not individually sufficient to establish cavity inclusion.

Grade C—limited or indirect molecular evidence. This grade was assigned when the interpretation relied predominantly on indirect or formulation-level observations, including FTIR, DSC, PXRD, morphology, phase-solubility behavior without spatial confirmation, gas-phase mass spectrometry, docking or other computational modeling, or biological/formulation performance without separate characterization of the binary vitamin–cyclodextrin interaction. Computational or gas-phase evidence alone was not considered sufficient to demonstrate the dominant solution-phase structure.

When individual techniques produced conflicting interpretations, classification was based on the strongest mutually consistent experimental evidence rather than on the most favorable individual result. Unresolved contradictions resulted in assignment of the more conservative grade. The included systems were reassessed according to these criteria, and borderline classifications were resolved by discussion among the evaluating authors until consensus was reached.

The evidence grade was considered separately from the mechanistic classification of the system. Thus, a vitamin could be categorized as undergoing true inclusion, partial inclusion, external association, physical entrapment, or covalent functionalization depending on its actual molecular role. Similarly, evidence for improved stability, release, permeability, cellular activity, or translational performance was evaluated independently and did not increase the molecular evidence grade.

## 3. Water-Soluble Vitamins: Structural and Physicochemical Considerations

The water-soluble vitamins constitute an unusually heterogeneous set of guest molecules. Thiamine contains a permanently charged thiazolium unit; riboflavin has a planar isoalloxazine chromophore attached to a polyhydroxylated ribityl chain; nicotinic acid, nicotinamide, pyridoxine, pyridoxal, and pyridoxal 5′-phosphate are substituted heteroaromatic compounds; pantothenic acid is a flexible aliphatic hydroxy acid; biotin contains fused ureido and sulfur-containing rings; folic acid combines pteridine, p-aminobenzoate, and glutamate fragments; vitamin B12 is a large metal-containing corrinoid; and ascorbic acid is an ionizable enediol lactone. These differences determine molecular size, conformational freedom, aromatic surface area, charge distribution, hydrogen-bonding capacity, and susceptibility to degradation.

For reference, the molecular formulas of the principal vitamin forms discussed in this review are: thiamine hydrochloride (C_12_H_18_Cl_2_N_4_OS), riboflavin (C_17_H_20_N_4_O_6_), nicotinic acid (C_6_H_5_NO_2_), nicotinamide (C_6_H_6_N_2_O), pantothenic acid (C_9_H_17_NO_5_), pyridoxine (C_8_H_11_NO_3_), pyridoxal (C_8_H_9_NO_3_), pyridoxal 5′-phosphate (C_8_H_10_NO_6_P), biotin (C_10_H_16_N_2_O_3_S), folic acid (C_19_H_19_N_7_O_6_), cyanocobalamin (C_63_H_88_CoN_14_O_14_P), and ascorbic acid (C_6_H_8_O_6_). The native α-, β-, and γ-cyclodextrins have the molecular formulas C_36_H_60_O_30_, C_42_H_70_O_35_, and C_48_H_80_O_40_, respectively. In contrast, chemically substituted cyclodextrins such as HPβCD and SBEβCD are typically mixtures differing in degree and distribution of substitution and therefore do not possess a single unique molecular formula.

High water solubility commonly arises from hydroxyl, amino, amide, phosphate, carboxylate, and other ionizable groups, but strong hydration can make cavity binding thermodynamically unfavorable. Complex formation requires compensation for the desolvation of both host and guest, and many vitamin molecules lack a sufficiently large nonpolar surface for deep inclusion. Their charge state is also pH-dependent. Nicotinic acid, folates, pantothenates, pyridoxal phosphates, and ascorbates may therefore exhibit different binding modes and apparent association constants depending on pH, buffer, counterions, and concentration. For amphiphilic vitamers, an aromatic or less polar fragment may enter the cavity while ionic and strongly hydrated groups remain at a cyclodextrin rim; for very large structures, only partial inclusion or external association is geometrically plausible.

The physicochemical limitations of these vitamins are not restricted to solubility. Thiamine and several B6 vitamers are temperature- and pH-sensitive, riboflavin is strongly photosensitive, folates undergo oxidative and photochemical degradation, cobalamins are affected by light and formulation composition, and ascorbic acid is readily oxidized. A recent multi-analyte stability study found pronounced instability of thiamine, pyridoxal, and ascorbic acid under commonly used short-term storage conditions, while riboflavin and 5-methyltetrahydrofolate also required careful temperature control [2]. Moreover, aqueous solubility does not guarantee high systemic exposure: absorption may be transporter-limited, degradation may occur before uptake, and excess water-soluble vitamin can be rapidly eliminated. Accordingly, cyclodextrin formulations should be evaluated in relation to the dominant limitation of each vitamer-chemical stability, permeability, residence time, compatibility, or release-rather than being justified by solubility alone.

## 4. Molecular Basis of Cyclodextrin–Vitamin Interactions and Its Biological Relevance

Cyclodextrins are cyclic α-(1→4)-linked glucooligosaccharides with a hydrophilic outer surface and a hydrated cavity that is less polar than bulk water. The natural α-, β-, and γ-cyclodextrins contain six, seven, and eight glucose units, respectively, and therefore provide cavities of increasing diameter. Hydroxypropylated, methylated, sulfobutylether, and other substituted derivatives modify aqueous solubility, charge, steric environment, aggregation, and compatibility with biological membranes and polymeric matrices. These features have supported the expansion of cyclodextrins from simple solubilizers to components of nanoparticles, hydrogels, polyrotaxanes, and responsive delivery systems [6,7,8,9,10].

Molecular recognition by cyclodextrins is therefore governed by complementarity between host and guest rather than by cavity size alone. For the vitamin, the principal determinants include molecular dimensions and shape, conformational flexibility, accessible aromatic or relatively nonpolar surface, charge and protonation state, hydrogen-bond donor and acceptor pattern, and hydration. For the cyclodextrin, recognition depends on cavity dimensions and polarity, the spatial arrangement of hydroxyl groups at the primary and secondary rims, host flexibility, and, for modified derivatives, the identity and distribution of substituents. A favorable complex therefore requires a balance between desolvation of the vitamin and cyclodextrin cavity and the stabilizing contributions of van der Waals contacts, hydrogen bonding, hydrophobic interactions, and, for charged species, electrostatic or ion–dipole interactions. Consequently, a strongly hydrated or ionized vitamin may bind only weakly despite an apparently suitable molecular size, whereas partial insertion of a less polar molecular fragment can still produce significant recognition when complemented by interactions at the cyclodextrin rims.

The conventional driving force for cyclodextrin complexation is replacement of energetically unfavorable cavity water by a suitably shaped nonpolar guest surface, together with van der Waals contacts and, where geometrically accessible, hydrogen bonding at the rims [15,16,17]. Hydrophilic guests can nevertheless associate when they contain a locally less polar ring or side chain, when only part of the molecule enters the cavity, or when electrostatic, ion–dipole, and hydrogen-bonding interactions compensate for the desolvation penalty. Complexes of water-soluble vitamins may therefore range from deep inclusion to shallow inclusion and external rim binding. In riboflavin systems, ROESY and molecular dynamics have demonstrated predominantly out-of-cavity hydrogen-bonded association under some conditions [18], whereas folate studies show that cavity participation depends strongly on cyclodextrin size and that gas-phase and solution-phase affinity estimates can diverge markedly [19].

Host selection cannot therefore be reduced to the rule that a larger cavity is better. A cavity that is too small may exclude the relevant molecular fragment, whereas an oversized cavity may provide weak contact and excessive mobility. For vitamin C, recent ion-mobility mass spectrometry and computational modeling identified β-cyclodextrin as the most compact fit among the native hosts, while also emphasizing that electrospray-generated adducts do not by themselves prove the dominant structure in solution [20]. Derivatization may improve practical performance without necessarily strengthening classical inclusion: HPβCD and SBEβCD can increase host solubility, reduce crystallization, introduce charge, and facilitate incorporation into biological or polymeric systems. For example, concentrated HPβCD and SBEβCD formulations have been used to increase riboflavin availability and corneal transport even though the molecular association may include substantial non-inclusion contributions [21].

For hydrophilic vitamins, evidence of interaction should thus be separated from evidence of cavity inclusion. Phase-solubility profiles, UV–visible or fluorescence changes, FTIR band shifts, DSC, PXRD, and altered morphology can support association or formation of a new solid phase, but they are rarely sufficient to establish guest orientation. Quantitative changes in the internal H3 and H5 cyclodextrin resonances, intermolecular ROESY/NOESY correlations, reliable ITC or NMR titrations, and agreement among orthogonal solution methods provide stronger support. Mass spectrometry and computational methods are valuable for composition and structural interpretation but require explicit consideration of protonation, solvation, aggregation, and gas-phase stabilization [7]. This distinction is essential for interpreting the individual vitamin systems discussed below and for linking molecular complexation to stability, release, and biological performance.

## 5. Cyclodextrin–Vitamin Systems: Molecular Interactions and Functional Consequences

Unlike fat-soluble vitamins [14], water-soluble vitamins do not generally require cyclodextrins primarily to overcome severe aqueous insolubility. Consequently, the literature on these compounds is more heterogeneous, and the objectives of cyclodextrin use extend beyond conventional solubilization. Depending on the vitamin and its chemical form, cyclodextrins have been employed to improve chemical or photochemical stability, alter release kinetics, construct polymeric or supramolecular carriers, facilitate analytical recognition, or provide a scaffold for targeting ligands. This broader functional spectrum also increases the risk of terminological overextension: many publications describe a “vitamin–cyclodextrin inclusion complex” even when the evidence supports only external association, physical entrapment in a cyclodextrin-containing matrix, or coexistence of the vitamin with a cyclodextrin complex of another guest. The following vitamin-by-vitamin analysis therefore considers preparation methods, evidence and mode of complexation, stoichiometry and stability constants, analytical and computational characterization, effects on stability and release, and the resulting pharmaceutical, food, cosmetic, and analytical applications.

For each system, functional outcomes related to chemical or formulation stability, release, permeability, biological activity, and translational validation are reported where available; absence of such evidence is treated explicitly as an evidence gap rather than inferred from molecular complexation or formulation performance.

The length and depth of the following vitamin-specific sections intentionally reflect the amount and quality of evidence available for each vitamin rather than an attempt to provide equal coverage. Vitamin C and riboflavin have comparatively extensive literature spanning molecular characterization, formulation, stability, release, and biological applications. By contrast, the evidence for pantothenate is limited, and the literature concerning biotin, folates, and cobalamins frequently involves cyclodextrin-containing materials in which the vitamin is not a directly characterized cavity guest.

Accordingly, systems involving covalent vitamin functionalization, physical entrapment, targeting functions, or use of the vitamin as a model permeant are discussed separately from directly characterized free-vitamin–cyclodextrin complexes. Their inclusion in this review is intended to clarify the actual molecular role of the vitamin and cyclodextrin and to prevent formulation-level or targeting effects from being interpreted as evidence of free-vitamin inclusion.

### 5.1. Thiamine (Vitamin B1)

The available literature on thiamine is centered mainly on thiamine hydrochloride and native α- and β-cyclodextrins. The most comprehensive solution and solid-state study prepared 1:1 thiamine hydrochloride complexes with αCD and βCD by solution mixing followed by cooling, filtration, washing, and drying. Job plots, UV–visible titrations, ESI-MS, DSC, FTIR, one-dimensional NMR, and 2D ROESY were combined to characterize the products [22]. A second approach used microwave irradiation to prepare a βCD–thiamine hydrochloride solid complex, followed by incorporation into chitosan–gelatin hydrogel networks [23]. These studies illustrate two common preparation strategies for water-soluble vitamin complexes: equilibrium association in aqueous solution followed by isolation, and energy-assisted solid-product preparation intended for subsequent loading into a polymeric carrier.

The evidence for inclusion is strongest in the αCD/βCD comparison because the authors obtained cavity-relevant ROESY correlations between thiamine protons and the H3/H5 protons of the cyclodextrins, while ESI-MS supported a 1:1 host–guest composition [22]. The spectral data were interpreted as dynamic participation of the pyrimidine and thiazolium portions of thiamine near or within the cavities, with stronger overall association for βCD. By contrast, the microwave-prepared βCD product was supported primarily by an A_L_-type phase-solubility profile, FTIR, DSC, PXRD, microscopy, and docking. These data establish interaction and a new solid-state environment, but the proposed orientation of the guest is less directly demonstrated than in the ROESY study [23].

Both studies proposed 1:1 complexes, but their quantitative constants differed markedly. UV–visible analysis at 298.15 K gave values of approximately 1.12–1.28 × 10^3^ M^−1^ for αCD and 1.67–1.90 × 10^3^ M^−1^ for βCD, depending on the absorption band and fitting approach [22]. The microwave/phase-solubility study reported an apparent stability constant of 122.437 ± 4 M^−1^ [23]. This difference should not be treated as a direct contradiction because the studies used different concentration ranges, analytical observables, sample histories, and mathematical models. It nevertheless demonstrates why association constants obtained by different methods should not be pooled or ranked without careful consideration of experimental conditions.

The principal pharmaceutical outcome was controlled release. Incorporation of the microwave-prepared βCD complex into chitosan–gelatin hydrogels reduced the rate of thiamine release relative to directly loaded vitamin and provided biodegradable matrices [23]. The αCD and βCD complexes were also examined in the presence of human serum albumin, demonstrating transfer or release of thiamine from the cyclodextrin environment [22]. In other work, βCD was used as a wall material in thiamine-containing microcapsules intended for lipid-rich systems, improving processing and storage stability, although microencapsulation in such a matrix should not automatically be equated with a discrete molecular inclusion complex [24]. Cyclodextrin-based ion-selective membranes have additionally been used for potentiometric determination of thiamine, but those sensors provide functional evidence of molecular recognition rather than full structural characterization of an isolated complex [25].

From a biological-delivery perspective, the principal relevance of cyclodextrin association with thiamine therefore lies in stabilization and modulation of vitamin release rather than in evidence for enhanced systemic bioavailability.

### 5.2. Riboflavin and Its Photoproducts (Vitamin B2)

Riboflavin provides one of the clearest examples of conflicting structural interpretations in the water-soluble-vitamin literature. Its planar isoalloxazine ring is a plausible hydrophobic binding element, whereas the ribityl chain is highly hydrated and can hinder deep penetration into a cyclodextrin cavity. Thermodynamic studies using solubility measurements and ^1^H NMR described 1:1 complexes with αCD and βCD. The αCD interaction was very weak, whereas βCD produced substantially larger apparent constants: approximately 38 ± 2 kg mol^−1^ from solubility measurements and 32 ± 1 kg mol^−1^ from NMR at 298 K. The proposed structure involved partial insertion of the hydrophobic ring system, while the ribityl substituent remained largely outside the cavity [26].

A later investigation reached a different conclusion. UV–visible spectroscopy, fluorescence, DSC, several NMR methods, DOSY, ROESY, and 50-ns molecular dynamics simulations were used to study riboflavin with βCD and HPβCD. The apparent phase-solubility constants were only 39.7 and 20.6 M^−1^, respectively. Importantly, the H3 and H5 signals of the cyclodextrins changed minimally, no diagnostic ROESY cross-peaks between riboflavin and cavity protons were detected, and the simulations favored “out-of-ring” hydrogen-bonded arrangements at the outer rim. The authors therefore classified the systems as non-inclusion complexes [18]. These results are more persuasive than conclusions based solely on increased solubility or fluorescence because they directly test spatial proximity to the cavity.

The two interpretations are not necessarily mutually exclusive under all conditions. Riboflavin may populate an ensemble containing external association, shallow inclusion, and aggregated species whose relative abundance depends on concentration, pH, host substitution, and analytical timescale. This possibility is supported by the modest binding energies and by the fact that different studies used substantially different host excesses and fitting assumptions. More recent βCD-containing nanocomposites reported a 1:1 A_L_-type phase-solubility profile and an apparent stability constant of 594.7 M^−1^, but the system was subsequently embedded in pectin–pullulan–kaolinite matrices and was characterized mainly by solid-state and spectroscopic methods. The quantitative result is useful for formulation development, but it does not by itself resolve the inclusion versus external-association question [27].

**Figure 2:**
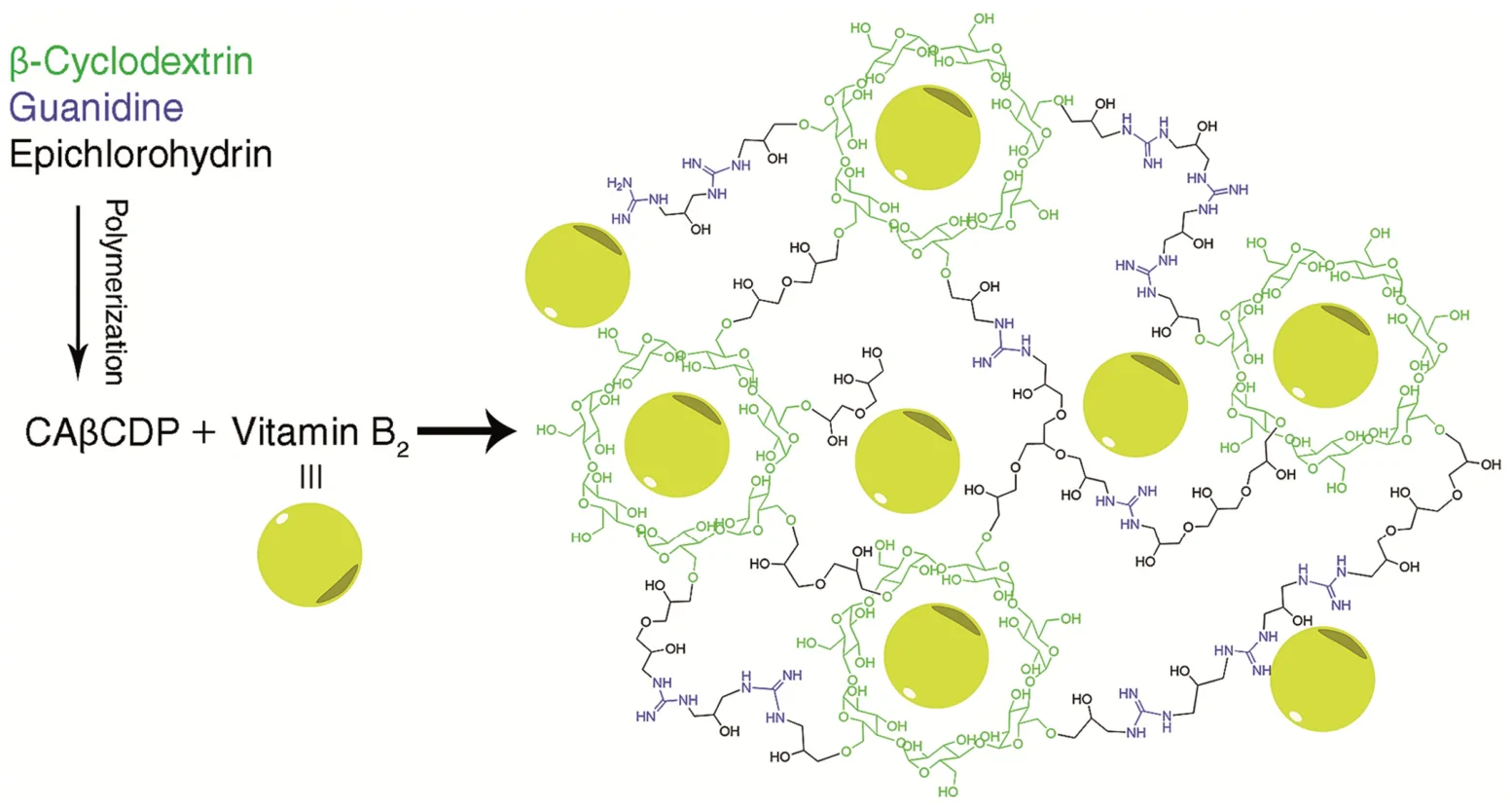
Vitamin B_2_ is effectively incorporated into novel water-soluble cationic β-cyclodextrin polymers in order to improve its physicochemical properties. Reproduced from [28] with permission from RSC.

Polymeric cyclodextrins have been investigated to obtain sustained riboflavin delivery (Figure 2). A water-soluble cationic poly(βCD-co-guanidine) was interpreted as accommodating part of the isoalloxazine system in cyclodextrin cavities while simultaneously binding additional riboflavin through electrostatic and non-inclusion interactions within the polymer network. Release was biphasic and dependent on pH and polymer composition [28]. Peracetylated βCD polymers and their electrospun nanofibers further slowed release, although DLS, zeta potential, FTIR, and thermal analysis cannot quantify what fraction of vitamin was truly cavity-bound [29]. Such studies are best described as cyclodextrin-based polymeric carriers containing both inclusion and non-inclusion binding sites.

Riboflavin photochemistry has also motivated hybrid liposomal formulations. In multicomponent liposomes, complexation with γCD was examined together with combinations of light absorbers and antioxidants. The resulting stabilization and entrapment were properties of the complete liposome–cyclodextrin–additive system rather than of the binary complex alone [30]. Conversely, a photochemical study of furaneol showed that βCD can increase, rather than decrease, a riboflavin-sensitized reaction rate. In that system, furaneol was the principal cavity guest, whereas riboflavin was proposed to associate externally with the cyclodextrin complex, bringing photosensitizer and substrate into proximity [31]. This is an important reminder that cyclodextrins do not invariably stabilize vitamins or surrounding formulation components; supramolecular organization can also facilitate degradation pathways.

The riboflavin photoproduct lumichrome is less hydrophilic and forms more readily quantifiable complexes. Phase-solubility studies indicated 1:1 complexes with HPβCD and HPγCD, with apparent constants of approximately 525 and 77 M^−1^, respectively. HPβCD increased lumichrome solubility more strongly and was selected for photocrosslinked collagen hydrogels. These materials were investigated for fibroblast-compatible matrices and for light-activated antibacterial effects against Staphylococcus aureus [32,33]. Lumichrome should, however, be discussed explicitly as a riboflavin derivative or photoproduct rather than as vitamin B2 itself.

Thus, for riboflavin, the biological significance of cyclodextrins appears to arise from modulation of stability, local concentration, release, and photochemical behavior, even when classical cavity inclusion is not the dominant interaction mechanism.

### 5.3. Nicotinic Acid and Nicotinamide (Vitamin B3)

Vitamin B3 comprises chemically and supramolecularly distinct vitamers. Nicotinic acid is amphoteric, and its predominant protonation state depends strongly on pH, whereas nicotinamide lacks the ionizable carboxyl group. The most informative experimental studies concern nicotinic acid and native cyclodextrins. ^1^H NMR and calorimetry indicated 1:1 association of the zwitterionic form with αCD and βCD at pH approximately 3.3–3.5. Surprisingly, αCD bound nicotinic acid more strongly than βCD: NMR constants were 23.4 ± 0.8 and 6.5 ± 0.5 kg mol^−1^, respectively. The αCD complex was strongly exothermic and entropically unfavorable, consistent with a tight, shallow fit, whereas βCD binding involved deeper inclusion, greater dehydration, and a more favorable entropy contribution [34].

Independent volume and heat-capacity measurements produced the same qualitative order, with approximate constants of 17 ± 5 kg mol^−1^ for αCD, 14 ± 2 kg mol^−1^ for HPαCD, and 5 ± 1 kg mol^−1^ for βCD; the HPβCD interaction was too weak for reliable quantification. Positive volume changes and negative heat-capacity changes were attributed to release of water and overlap of hydration shells during association [35]. The preference for αCD demonstrates that the largest available cavity is not necessarily the best host: a smaller cavity can yield a more favorable balance of contact, hydrogen bonding, and dehydration when the guest is compact.

A broader spectroscopic and calorimetric study of nicotinic acid with βCD reported substantially larger constants. Job plots, surface tension, conductivity, UV–visible spectroscopy, 2D ROESY, and ITC supported a 1:1 complex in which the pyridine ring entered the cavity. At 298 K, nonlinear UV analysis gave approximately 1.23 × 10^3^ M^−1^ and ITC gave (1.498 ± 0.155) × 10^3^ M^−1^, with negative enthalpy and entropy changes [36]. These values exceed the earlier NMR/calorimetric data by almost two orders of magnitude. The discrepancy likely reflects differences in speciation assignments, ionic conditions, concentration windows, and data treatment. In particular, the earlier study explicitly calculated that the zwitterion exceeded 98% under its conditions, whereas the later paper described a closely related pH range using a different ionic interpretation. For a critical review, both data sets should be retained rather than averaged.

Computational studies complement these experiments but also demonstrate the sensitivity of predicted geometry to protonation state. DFT calculations for zwitterionic nicotinic acid emphasized dispersion, electrostatic interactions, and hydrogen bonding in a hydrated βCD complex [37]. A separate study of neutral nicotinic acid favored an orientation with the pyridine ring inside βCD and the carboxyl group near the primary rim [38]. These models are chemically plausible, but neither establishes which state and orientation dominate in experimental solution. Future modeling should treat pH-dependent speciation, explicit water, and transitions between external and included states rather than optimizing only a few preselected geometries.

Nicotinamide has been studied mainly in multicomponent formulations. HPβCD-containing Eudragit S100 nanoemulsified particles provided high loading, sustained release, and prolonged antimicrobial action, but no separate binding constant, stoichiometry, or cavity-specific NMR evidence was reported. The material is therefore more accurately described as an HPβCD-containing polymeric delivery system for nicotinamide than as a classical isolated inclusion complex [39]. Likewise, ZnO/βCD/nicotinic-acid nanocomposites have been evaluated for photocatalytic, antimicrobial, and cytotoxic activity, but their multicomponent crystalline architecture does not permit a simple assignment of nicotinic acid as a discrete βCD guest [40].

### 5.4. Pantothenic Acid and Pantothenate Salts (Vitamin B5): Limited Molecular Evidence

The B5 literature is sparse and focuses on salts rather than un-ionized pantothenic acid. Electrospun polymer-free nanofibers were prepared from HPβCD and a commercial DL-pantothenic acid hemicalcium salt. The host and guest were mixed in a nominal 1:1 molar ratio in concentrated aqueous HPβCD solutions and processed by electrospinning. An A_L_-type phase-solubility diagram gave an apparent constant of 214.46 M^−1^, and FTIR, PXRD, thermal analysis, microscopy, and docking were interpreted as evidence of inclusion [41]. Because no NMR, ROESY, or calorimetric binding study was performed, the data establish a new supramolecular solid and improved formulation behavior more convincingly than they establish a unique guest orientation inside the cavity.

The nanofibers showed improved thermal behavior of the pantothenate component and strong antibacterial activity against Escherichia coli and Staphylococcus aureus [41]. The reported cancer-cell results require caution: at the highest tested concentration, the HPβCD/pantothenate nanofibers produced greater HCT-116 viability than either free pantothenate or HPβCD, which is inconsistent with the paper’s description of enhanced anticancer activity. The result may instead indicate reduced cytotoxicity after formulation. This example highlights the need to distinguish delivery-related changes in exposure from true enhancement of a biological effect.

A solution study of calcium D-pantothenate with βCD and HPβCD used density, sound velocity, compressibility, conductivity, and ^1^H NMR over a range of temperatures. The largest NMR changes involved H3 and H5, consistent with some penetration of the cavity, but external proton shifts and the thermodynamic observables indicated extensive hydrophilic and ion–dipole interactions at the cyclodextrin rims. βCD interacted more strongly than HPβCD, yet no stability constant or independently established stoichiometry was reported; the NMR samples were simply prepared at 1:1 composition [42]. The most defensible interpretation is therefore partial inclusion accompanied by substantial external association and hydration changes.

The vitamin B5 studies therefore demonstrate formulation-dependent changes in release and biological responses, but do not yet establish that molecular inclusion itself is responsible for these effects.

### 5.5. Pyridoxine, Pyridoxal, and Pyridoxal 5′-Phosphate (Vitamin B6)

Vitamin B6 provides a useful illustration of vitamer-dependent cyclodextrin binding. Pyridoxine, pyridoxal, and pyridoxal 5′-phosphate (PLP) differ in charge, hydrogen-bonding capacity, and chemical reactivity, and the reported constants span almost two orders of magnitude. Pyridoxine–βCD solids have been prepared by kneading, co-precipitation, and freeze-drying. FTIR, PXRD, DSC, ^1^H NMR, and UV–visible spectroscopy supported interaction and altered solid-state organization, with the kneaded product reported as the most extensively transformed. However, the 1:1 ratio was imposed during preparation and neither a stability constant nor 2D NMR evidence was provided [43].

A competitive fluorescence method estimated a much weaker interaction between an unspecified “vitamin B6” sample and HPβCD. Displacement of fluorescent dyes from HPβCD was analyzed using a 1:1 model, yielding 71 ± 4 L mol^−1^ [44]. The approach is analytically useful but indirect: it confirms competition for a cyclodextrin-associated environment without identifying the exact vitamer or proving a single cavity geometry. The same study used the competitive response for determination of vitamin B6 in tablets and injections.

PLP produced the highest reported B6 binding constant. A binary PLP–βCD solid was prepared by kneading equimolar components. Fluorescence analysis gave a 1:1 complex and K = 5772.3 M^−1^, while ^1^H NMR, FTIR, LC-MS, and docking supported close association. The phosphate group was modeled near the hydroxymethyl rim while the heteroaromatic part occupied the cavity [45]. The complex was then displayed on βCD-stabilized silver nanoparticles and used as a fluorescent sensor for hydrazine, with a detection limit of 0.513 μM. Although the nanomaterial adds additional interactions, the separate characterization of the PLP–βCD pair makes this one of the better-supported B6 systems.

Pyridoxal was similarly employed in an analytical displacement platform. Fluorescence fitting gave a 1:1 pyridoxal–βCD association constant of 3421.6 M^−1^, and FTIR plus DFT supported a cavity-bound model [46]. The absence of NMR or ROESY means that the spatial assignment remains less direct than for the PLP system. Folic acid was then used to displace pyridoxal from βCD-functionalized copper nanoclusters, producing an analytical response. Importantly, the mechanistic explanation relied on a folate constant taken from ESI-MS and therefore overestimated the solution affinity of folate; this issue is discussed below under vitamin B9 and in the methodological section.

Several additional B6 sensors use cyclodextrin-modified nanomaterials without separately characterizing a binary vitamin complex. βCD-functionalized ZnO quantum dots containing PLP or pyridoxal were used for histamine detection [47], and βCD-modified nitrogen-doped graphene quantum dots containing pyridoxal were used for dopamine sensing [48]. βCD-based potentiometric membranes and βCD/Zr-doped ZnO carbon electrodes were developed for pyridoxine analysis [25,49]. These are important applications of cyclodextrin-mediated recognition, but they should not be cited as structural proof of isolated B6 inclusion complexes. A biomimetic PLP-dependent transamination system likewise used βCD to bind the keto-acid substrate rather than PLP itself; its relevance is to cyclodextrin-assisted mimicry of B6-dependent enzymes, not to a PLP⊂CD complex [50].

### 5.6. Biotin and Biotin-Functionalized Cyclodextrin Systems (Vitamin B7)

The available Vitamin B7-CD literature does not provide convincing evidence for a classical binary complex in which free biotin is included in a native cyclodextrin cavity. Instead, biotin is used predominantly as a covalently attached targeting ligand. Cyclodextrin dimers linked through glutamic-acid-based spacers and functionalized with biotin were designed to bind doxorubicin cooperatively in two cavities while directing the carrier toward the sodium-dependent multivitamin transporter. The biotinylated βCD dimer increased the activity of doxorubicin against MCF-7 cells, but the cavity guest was doxorubicin, and the vitamin was part of the host architecture [51].

The same conceptual distinction applies to HPβCD-based redox-responsive micelles in which biotin was esterified to the cyclodextrin scaffold, a disulfide-linked aggregation-induced-emission unit formed the hydrophobic core, and paclitaxel was loaded as the therapeutic cargo [52]. Biotinylated βCD has also been used to coat Bi2O3 nanoparticles for antimicrobial and anticancer studies [53]. In both cases, spectroscopic evidence confirms covalent attachment of biotin, not its inclusion.

A different architecture used a biotin-functionalized BODIPY as a larger guest for cationic βCD. The conjugate and βCD assembled into two-dimensional nanosheets, subsequently loaded with a hemin/G-quadruplex catalytic system for combined photodynamic and chemodynamic therapy [54]. Here biotin is part of the guest molecule, but the publication does not establish that the biotin moiety itself is the cavity-binding segment; the hydrophobic BODIPY group is the more plausible driver of association. The B7 evidence base therefore supports a subsection on biotin-functionalized cyclodextrin systems, while simultaneously demonstrating the need to avoid presenting such constructs as complexes of free vitamin B7.

### 5.7. Folic Acid, Folates, and Folate-Functionalized Systems (Vitamin B9)

The most rigorous B9 study compared sodium folate with all three native cyclodextrins by ^1^H NMR, ITC, and ESI-MS, while explicitly accounting for folate self-association. The dominant composition was approximately 1:1. NMR gave association constants below 5 M^−1^ for αCD, 118 ± 11 M^−1^ for βCD, and 17 ± 2 M^−1^ for γCD. ITC gave 162 ± 13 M^−1^ for βCD in water, 136 ± 11 M^−1^ in HEPES buffer at pH 7.4, and 56 ± 26 M^−1^ for γCD. The αCD heat effect was too small for reliable fitting [19].

The NMR shifts indicated different modes of association. For βCD and γCD, the largest changes involved H3 and H5 and the aromatic folate protons, consistent with cavity participation. For αCD, the largest host change occurred at the external H6 position, supporting an exclusion complex in which the cavity was too small to accommodate the guest. The order αCD < γCD < βCD therefore reflected cavity fit rather than a simple increase with cavity diameter. The authors also measured a sodium-folate dimerization constant of 6.6 ± 0.3 and included self-association in the binding analysis, an important methodological strength [19].

The same work exposed a major limitation of ESI-MS-derived “association constants.” ESI-MS yielded apparent values of 6.90 × 10^3^, 14.81 × 10^3^, and 9.32 × 10^3^ M^−1^ for αCD, βCD, and γCD, respectively, which are orders of magnitude above the NMR and ITC results. The authors attributed this to loss of hydrophobic stabilization and enhancement of electrostatic interactions during transfer to the gas phase [19]. Consequently, the frequently quoted value 14,810 M^−1^ should not be interpreted as the solution binding constant of folate with βCD.

This distinction is directly relevant to the βCD–copper-nanocluster sensor based on pyridoxal displacement. That paper compared a pyridoxal–βCD fluorescence constant of 3421.6 M^−1^ with the ESI-MS folate value of 14,810 M^−1^ to rationalize displacement [46]. The comparison combines different phases and methods and is therefore not quantitatively valid. Folate may still displace pyridoxal in the specific nanoparticle environment because of concentrations, surface interactions, aggregation, or photophysical effects, but the mechanism cannot be justified by the quoted constants alone.

Folic acid has also been loaded into βCD-containing chitosan/collagen scaffolds for neuronal tissue engineering. The reported βCD/folic-acid ratio was 2:1 by mass rather than by mole, and the binary complex was not isolated or characterized separately. FTIR of the multicomponent scaffold, improved thermal behavior, prolonged release over several days, and favorable fibroblast and Neuro-2A responses demonstrated a useful delivery material but only weak evidence for molecular inclusion [55]. In a separate and extensive field, folate is covalently appended to cyclodextrins as a receptor-targeting ligand while the cavity carries another drug or remains available for membrane interactions. Such systems should be categorized as folate-functionalized cyclodextrins, not folate inclusion complexes.

For folates, the distinction between molecular inclusion, matrix-assisted delivery, and covalent targeting is particularly important because these mechanisms have fundamentally different implications for biological availability and cellular targeting.

### 5.8. Cobalamins in Cyclodextrin-Containing Delivery Systems (Vitamin B12)

The cobalamins are much larger than the cavities of individual native cyclodextrins, and the reviewed literature contains no convincing example of a classical complex in which the complete vitamin B12 molecule is included in one cavity. The most relevant studies instead use vitamin B12 as a hydrophilic model cargo in supramolecular hydrogels or as a probe of transdermal transport. This distinction should be made explicit because the term “cyclodextrin–vitamin B12 system” can otherwise imply a molecular host–guest structure that was not demonstrated.

In αCD/PLGA–PEG–PLGA hydrogels, the actual inclusion event is threading of αCD onto PEG blocks, while hydrophobic PLGA domains provide additional physical crosslinks. Vitamin B12 is dissolved in the precursor solution and physically trapped in the resulting network. Release was highly prolonged, with only approximately 20–35% of the loaded vitamin released over about 700 h, and the kinetic models indicated contributions from diffusion and matrix erosion [56]. No B12–αCD binding constant, stoichiometry, or cavity-specific NMR evidence was reported.

A related αCD/PCL–PEG–PCL hydrogel used the same poly(pseudo)rotaxane principle. The results section reported only approximately 25–35% B12 release after about 600 h, whereas the abstract stated release up to 80% within 20 days, an internal inconsistency that should be acknowledged if quantitative values are cited [57]. The authors attributed slow release partly to the molecular size and hydrogen-bonding capacity of B12, but those interactions could occur throughout the hydrated polymer network and do not demonstrate penetration of a cyclodextrin cavity.

HPβCD grafted to polyethylenimine was developed as a skin-penetration enhancer for vitamin B12. The most effective HPβCD–PEI material increased transdermal transport approximately sixfold relative to control and outperformed Azone [58]. A zwitterionic HPβCD–PEI–phosphorylcholine derivative subsequently increased B12 flux by roughly 20- to 40-fold, depending on concentration [59]. Mechanistic studies implicated disruption or reorganization of stratum-corneum keratin and lipids. In both cases, B12 served as a large hydrophilic model permeant; no evidence showed that it occupied HPβCD cavities. The appropriate category is therefore a cyclodextrin-based penetration enhancer rather than a B12 inclusion complex.

Consequently, the biological relevance of cyclodextrins in the available B12 systems is primarily related to carrier architecture and barrier modulation rather than direct molecular encapsulation of the vitamin.

### 5.9. Ascorbic Acid, Ascorbates, and Vitamin C Derivatives

Vitamin C has generated by far the largest and most diverse literature among water-soluble vitamins. The chemical instability of L-ascorbic acid and ascorbate toward oxygen, light, heat, metals, and pH changes provides a clear rationale for protective formulation. Nevertheless, the small, highly polar guest produces only modest or method-dependent binding in many systems, and the literature ranges from quantitatively studied binary complexes to food matrices in which cyclodextrin and vitamin C merely coexist. The evidence must therefore be separated into direct host–guest studies, solid-state formulations, hybrid delivery systems, food applications, and analytical platforms.

A foundational study prepared an equimolar sodium ascorbate–αCD complex by stirring under nitrogen in the dark followed by freeze-drying. A kinetically derived apparent constant of 112 M^−1^ was obtained from the concentration-dependent slowing of photodegradation, assuming a 1:1 model, and DSC supported formation of a new solid-state product [60]. The constant should be described as an apparent kinetic value rather than a directly measured equilibrium constant. Complexation alone increased the photochemical half-life only from approximately 0.9 to 2 h. The widely cited 120-fold stabilization, to approximately 112 h, required the complete combination of αCD complexation, multilamellar liposomal entrapment, and light absorbers in the aqueous and lipid phases [60]. Later factorial-design studies confirmed that improved photostability was often accompanied by reduced liposomal entrapment of the complex, demonstrating a formulation-level trade-off rather than an unqualified benefit.

The strongest direct solution evidence for L-ascorbic acid with βCD was provided by a study combining Job plots, UV–visible spectroscopy, surface tension, conductivity, 2D ROESY, and ITC. The data supported a 1:1 complex. At 298 K, nonlinear UV fitting gave approximately 3.06 × 10^3^ M^−1^, whereas ITC gave (3.655 ± 0.335) × 10^3^ M^−1^ with negative enthalpy and entropy changes. ROESY correlations between ascorbic-acid protons and βCD H3/H5 were interpreted as cavity inclusion [36]. These values are substantially higher than the sodium-ascorbate/αCD kinetic constant and emphasize the combined influence of vitamin form, host size, pH, ionic state, and analytical method.

Several solid-state studies used nominal 1:1 compositions without independently determining equilibrium stoichiometry. Kneading, co-precipitation, and freeze-drying of L-ascorbic acid with βCD produced altered FTIR, PXRD, DSC, ^1^H NMR, and UV–visible profiles. Co-precipitation and freeze-drying appeared more effective than kneading, but no stability constant, quantitative chemical-shift table, or 2D NMR data were provided [61]. High hydrostatic pressure was also used to prepare βCD/ascorbic-acid solids. The highest product loading was reached near 300 MPa, but the reported “inclusion ratio” represented vitamin content relative to a theoretical 1:1 maximum and reached only about 50%; it did not prove that half of all molecules were structurally included. FTIR, UV–visible, and DSC supported interaction but not the detailed orientation proposed by the authors [62].

Trapped ion-mobility mass spectrometry and computational modeling have more recently compared 1:1 ions of L-ascorbic acid with αCD, βCD, and γCD. βCD produced the most compact collision-cross-section change and the most favorable calculated cavity fit, whereas γCD ions showed the greatest gas-phase resistance to collision-induced dissociation. The work is valuable for comparing ion conformations, but it did not determine solution binding constants and explicitly acknowledged that electrospray can preserve nonspecific adducts [20]. The folate precedent shows why gas-phase stability and solution affinity should not be treated as interchangeable.

Electrospun and topical materials represent a major application area. A βCD/ascorbic-acid product prepared at nominal 1:1 composition was immobilized on cellulose acetate and deposited onto PVA nanofibers. The composite changed a burst-release profile into near-zero-order release, reported as approximately 8.2 mg g^−1^ h^−1^, although the paper contained an inconsistency between an 8-h experimental window and a statement referring to 48 h [63]. PVA nanofibers have also been prepared by directly adding a nominal 1:1 βCD/vitamin-C solution to the polymer. UV–visible and FTIR changes were used to claim inclusion, but the binary complex was not isolated, and no release or stability study was conducted [64].

HPβCD/ascorbic-acid solids have been incorporated into κ-carrageenan/PLA nanofibers for blueberry preservation. The preparation used a 1:2 host-to-vitamin feed ratio, which was not an experimentally established complex stoichiometry. FTIR, PXRD, and thermal analysis showed amorphization and interaction, while the nanofibers provided slow recovery of measurable vitamin and antioxidant activity [65]. The reported decline in “cumulative release” after an initial maximum indicates that chemical degradation occurred during the assay; the experiment therefore measured the balance of release and vitamin loss rather than release alone.

SBEβCD has served simultaneously as a potential host and as an ionic crosslinker in chitosan nanoparticles containing vitamins C and E. The vitamin/SBEβCD mixtures were pre-equilibrated at nominally equimolar composition before ionic gelation. Vitamin C association efficiency was approximately 78%, and about 52% was released over seven days. XPS suggested that the vitamin was not concentrated at the external surface, while FTIR indicated extensive hydrogen bonding with the polymeric matrix [66]. Because the binary pair was not characterized separately, this system is best described as vitamin-C-loaded SBEβCD-crosslinked chitosan nanoparticles rather than an unequivocal isolated inclusion complex.

Lipophilic vitamin C derivatives show a different binding regime. Ascorbyl tetraisopalmitate was combined with γCD at a feed composition close to two cyclodextrins per guest. PXRD and DSC indicated formation of an amorphous product containing 29.8% derivative, stable for one year under the tested conditions, and release in ethanol was faster than from the uncomplexed derivative [67]. The large, multi-chain guest is unlikely to be fully contained in one cavity; partial or multivalent inclusion of aliphatic chains by more than one γCD is more plausible. Similarly, an ascorbate–tocopherol phosphodiester conjugate formed 1:1 and 1:2 complexes with HPβCD, but binding was driven predominantly by the hydrophobic tocopherol portion rather than by the ascorbate moiety.

Food applications provide mixed evidence for stabilization. Direct addition of 1.5% βCD to pasteurized orange juice or ultra-frozen mandarin juice did not significantly improve vitamin C retention [68]. In contrast, 1% HPβCD modestly improved vitamin C retention and antioxidant quality in a mandarin juice enriched with pomegranate and goji components, although it also reduced fresh-aroma intensity, presumably by binding volatile compounds [69]. In preserved pepper, βCD reduced vitamin C loss during storage, but the proposed mechanism was indirect protection of phenolics and the broader antioxidant network rather than direct ascorbic-acid inclusion [70]. These studies show that effects observed in foods may arise from matrix-level sequestration of pro-oxidants, phenolics, aromas, or oxygen-sensitive components and cannot be assigned to a binary vitamin complex without targeted analysis.

Cyclodextrin-based analytical systems use several different mechanisms. HPβCD improved capillary-electrophoretic separation of ascorbate and preservatives through transient association and changes in effective electrophoretic mobility [71]. A βCD/CNT electrode enabled simultaneous detection of favipiravir, paracetamol, and vitamin C, with a vitamin-C detection limit of 0.21 μM; FTIR was used to propose a cavity-bound model, but no NMR or binding constant was obtained [72]. In Fe–βCD nanozymes, ascorbic acid acted as a reductant that suppressed TMB oxidation, while βCD participated in the iron-containing catalytic material; this is an analytical reaction rather than a vitamin inclusion complex. Such examples are valuable applications of cyclodextrin-containing interfaces, but they should be placed in a separate analytical table rather than used as structural evidence.

Overall, the vitamin C literature demonstrates both the usefulness and the interpretive risk of cyclodextrin formulation. The best-supported binary systems combine quantitative solution methods with cavity-specific NMR. Many highly functional materials, however, achieve stabilization or controlled release through a combination of inclusion, hydrogen bonding, polymer entrapment, diffusion barriers, and indirect matrix effects. Their technological value does not depend on calling every interaction an inclusion complex, and more precise terminology would strengthen rather than diminish the reported applications (Table 2 and Table 3).

## 6. Molecular Determinants of Stability, Delivery, and Biological Performance

### 6.1. Preparation Methods and Formulation Strategies

The preparation methods used for water-soluble vitamins can be divided into four broad groups. The first comprises equilibrium solution methods followed by isolation, including co-precipitation, cooling crystallization, evaporation, and freeze-drying. These methods were used for thiamine, pyridoxine, sodium ascorbate, and several native-CD complexes and are best suited to studies that also characterize the association in solution. The second group comprises mechanical or energy-assisted methods such as kneading, microwave irradiation, sonication, high hydrostatic pressure, and electrospinning. These approaches can increase production efficiency or facilitate direct conversion into a dosage form, but the resulting loss of crystallinity or altered thermal profile may reflect amorphization and intimate mixing as well as inclusion. The third group comprises polymeric cyclodextrins, cyclodextrin-functionalized nanoparticles, and poly(pseudo)rotaxane hydrogels. These materials contain multiple possible binding environments and should be analyzed as networks rather than as collections of identical 1:1 cavities. The fourth group comprises in situ analytical interfaces, in which a cyclodextrin is immobilized on an electrode or nanoparticle and the vitamin is recognized transiently during measurement.

Across the vitamin series, no single preparation method consistently produced the strongest or most rigorously demonstrated complex. Microwave preparation gave a practical thiamine product but a lower apparent constant than solution UV analysis [22,23]. For ascorbic acid, co-precipitation and freeze-drying produced more pronounced solid-state changes than kneading, yet none of these methods independently established the equilibrium composition [61]. Energy-assisted or scalable processing should therefore be evaluated separately from molecular characterization: a preparation can be technologically useful even when the cavity occupancy remains uncertain.

### 6.2. Cavity Size, Vitamer Structure, and Speciation

Host preference is governed by fit and hydration rather than by cavity size alone. Nicotinic acid bound αCD more strongly than βCD in the NMR/calorimetric work because the smaller cavity provided a tight shallow fit [34]. Riboflavin displayed the opposite trend because αCD was too small for effective contact with the isoalloxazine system, while βCD permitted partial insertion [26]. Folate showed a still different pattern: αCD formed an exclusion complex, βCD gave the strongest solution association, and γCD was too large for optimal stabilization [19]. These examples argue against universal claims that βCD or a hydroxypropyl derivative is always the preferred host.

Vitamer identity and protonation are equally important. PLP and pyridoxal gave much larger B6 constants than the weak competitive value reported for an unspecified vitamin B6 sample [44,45,46]. Nicotinic acid calculations produced different preferred structures for neutral and zwitterionic states [37,38]. Pantothenic-acid studies actually used calcium salts, and the folate study used sodium folate. Explicit reporting of chemical form, pH, buffer, ionic strength, and counterions is therefore essential. A review table should not collapse free acids, salts, phosphorylated cofactors, and covalent derivatives into a single generic vitamin entry.

### 6.3. Association Constants and Their Comparability

Reported constants range from below 5 M^−1^ for the αCD–folate exclusion system to several thousand M^−1^ for βCD complexes of PLP, pyridoxal, ascorbic acid, and nicotinic acid. The numerical spread is partly chemical, but method dependence is equally important. For nicotinic acid, one set of NMR and calorimetric experiments gave constants in the single- to tens-of-kg-mol^−1^ range, whereas a later UV/ITC study gave approximately 1.5 × 10^3^ M^−1^ [34,36]. Thiamine βCD values differed by more than an order of magnitude between phase-solubility and UV approaches [22,23]. Folate ESI-MS values exceeded NMR/ITC constants by roughly two orders of magnitude [19]. Importantly, molecular binding strength should not be equated directly with chemical stability of the vitamin. A higher association constant indicates a larger bound fraction under defined equilibrium conditions, but improved resistance to photochemical, oxidative, thermal, or other degradation processes must be demonstrated independently by stability-specific experiments.

Accordingly, association constants should be compared only when the vitamin form, solvent composition, temperature, pH, concentration range, stoichiometric model, and analytical phase are sufficiently similar. Constants expressed on a molality basis are not numerically interchangeable with molarity-based values. Apparent phase-solubility constants may include aggregation and non-inclusion solubilization, while kinetic protection constants measure the functional consequence of association under a degradation experiment. The preferred approach is nonlinear global fitting of multiple observables, supported by an independent method such as ITC or NMR and accompanied by uncertainty estimates and model comparison.

### 6.4. From Molecular Association to Stability, Bioavailability, and Biological Effects

The most reproducible technological effects are altered chemical stability and release. Sodium ascorbate photoprotection, lumichrome photochemical delivery, thiamine hydrogel release, pantothenate nanofibers, riboflavin polymeric carriers, and B12-loaded supramolecular hydrogels all demonstrate that cyclodextrin-containing systems can substantially change the exposure profile of a vitamin [23,29,33,56,60]. In many cases, however, release is governed by the polymeric matrix, fiber hydration, hydrogel erosion, or nanoparticle diffusion rather than by dissociation of a single host–guest pair.

Claims concerning bioavailability are less well supported. Most publications infer improved bioavailability from increased solubility, controlled release, or enhanced membrane transport rather than measuring systemic exposure in a pharmacokinetic study. The B12 transdermal work is a useful example: substantial flux enhancement was demonstrated, but the mechanism involved perturbation of the skin barrier by HPβCD-based polymers rather than formation of a B12 inclusion complex [58,59]. Similarly, improved cell responses in folate scaffolds or biotin-targeted carriers arise from the full material architecture and cannot be attributed solely to cavity binding.

Biological activity can increase, decrease, or change qualitatively after formulation. Riboflavin–CD associations enhanced solubility and prostate-cancer-cell effects even when the interaction was non-inclusion [18]. Cyclodextrin organization accelerated riboflavin-sensitized furaneol oxidation [31]. Pantothenate nanofibers showed antibacterial effects but not the claimed increase in cancer-cell toxicity [41]. Consequently, biological outcomes should be interpreted using exposure-matched controls for the free vitamin, free cyclodextrin, physical mixture, empty carrier, and, where applicable, a non-complexing polymer control.

To visualize the progression from molecular interaction to biological validation, the principal systems discussed in this review were additionally mapped against successive levels of evidence (Table 4). The purpose of this analysis is not to equate heterogeneous endpoints, but to identify how far each system has progressed along the molecular-to-translational evidence chain. Importantly, evidence at one level was not considered proof of the subsequent level; for example, altered release was not classified as enhanced permeability, and enhanced permeability was not classified as improved systemic bioavailability.

The matrix reveals a pronounced narrowing of the evidence base as biological relevance increases. Molecular association has been characterized for several vitamin–cyclodextrin pairs, and multiple formulations demonstrate improved stability or controlled release. Permeability enhancement has been demonstrated in selected systems, particularly riboflavin corneal delivery and B12 transdermal transport. Cellular or other biological effects have been examined only in a limited subset and are frequently attributable to the complete formulation rather than to cavity binding alone. Most importantly, the reviewed evidence does not establish improved systemic pharmacokinetic exposure to a free water-soluble vitamin as a direct consequence of cyclodextrin complexation, and no clinically validated benefit could be assigned specifically to a water-soluble vitamin–cyclodextrin interaction.

### 6.5. Biological Fate and Physiological Consequences of Cyclodextrin–Vitamin Systems

The biological consequences of cyclodextrin association extend beyond changes in apparent solubility or release kinetics. For water-soluble vitamins, the relevant biological sequence includes preservation of the chemically intact vitamin before administration, release from the carrier, access to an epithelial or tissue surface, membrane transport, cellular uptake, and ultimately participation in vitamin-specific metabolic or coenzyme-dependent processes. Importantly, evidence supporting one step of this sequence should not be interpreted automatically as evidence for improvement at subsequent levels.

#### 6.5.1. Permeability, Uptake, and Transporter-Related Processes

Among the reviewed systems, the most direct evidence for an effect on biological transport concerns permeability rather than systemic absorption. HPβCD-based polymeric penetration enhancers substantially increased transdermal transport of vitamin B12, with reported increases of approximately sixfold and, for a zwitterionic derivative, approximately 20–40-fold. However, these effects were attributed primarily to changes in the skin barrier caused by the cyclodextrin-containing polymers rather than to molecular inclusion of vitamin B12 itself. Thus, enhanced permeability in this case represents a formulation-level biological effect rather than evidence that formation of a vitamin–cyclodextrin complex improves uptake.

A related example is riboflavin, for which cyclodextrin derivatives have been used to increase availability at the corneal surface and facilitate transepithelial delivery. Again, the contribution of classical cavity inclusion cannot always be separated from effects of cyclodextrin concentration, substitution, hydration, and interactions with epithelial barriers. These examples illustrate why permeability enhancement should be experimentally distinguished from host–guest complexation.

Direct evidence that cyclodextrin binding modifies recognition of water-soluble vitamins by their physiological transporters is currently scarce. This is an important limitation because several vitamins are absorbed through saturable carrier-mediated pathways. A vitamin retained within a sufficiently stable cyclodextrin complex may not necessarily be recognized by its transporter until dissociation occurs. Conversely, rapid reversible association may protect a vitamin before reaching the absorption site while still allowing release close to the transporter. These possibilities remain largely hypothetical for the systems reviewed here because comparative transporter-specific uptake studies of free and cyclodextrin-associated vitamins are generally lacking.

#### 6.5.2. Biological Activity and Cellular Responses

Cyclodextrin-containing formulations can modify biological activity, but attribution of the effect requires appropriate controls. Riboflavin–cyclodextrin systems have been associated with changes in cellular effects even when structural studies favored predominantly non-inclusion association. Pantothenate-containing HPβCD nanofibers showed antibacterial activity, whereas the reported cancer-cell results did not support a straightforward enhancement of cytotoxicity. Folic-acid-loaded cyclodextrin-containing scaffolds produced favorable cellular responses in tissue-engineering models, but these effects reflected the complete multicomponent material and cannot be assigned specifically to molecular folate inclusion.

These examples demonstrate that an altered biological response may result from changes in local concentration, release kinetics, matrix interactions, cellular exposure, or the independent biological properties of the cyclodextrin-containing carrier. Consequently, comparisons should ideally include free vitamin, free cyclodextrin, a physical host–guest mixture, the empty delivery system, and the complete formulation.

#### 6.5.3. Metabolism and Intracellular Availability

Evidence that cyclodextrin association directly alters the metabolism of water-soluble vitamins is currently very limited. Most studies terminate at physicochemical characterization, release testing, permeability measurements, or short-term cellular assays and do not follow the vitamin after cellular entry. Therefore, it remains unclear whether cyclodextrin-mediated delivery changes intracellular conversion to active coenzyme forms, metabolic turnover, tissue retention, or elimination.

This distinction is especially important because preservation of vitamin content during storage or increased delivery across a biological barrier does not necessarily imply greater intracellular biological activity. Future studies should therefore quantify not only the parent vitamin but, where relevant, its active metabolites or coenzyme forms in cells and tissues.

#### 6.5.4. Toxicity and Biological Attribution

The biological effects of cyclodextrin-containing formulations must also be interpreted in the context of the carrier itself. Changes in membrane permeability, cell viability, or tissue penetration may arise from the cyclodextrin derivative or from the polymeric architecture independently of the vitamin. This is particularly relevant for systems designed intentionally to perturb biological barriers. The biological fate of a cyclodextrin-associated vitamin therefore depends not only on whether molecular association occurs, but also on when and where the complex dissociates relative to the relevant biological barrier, transporter, or intracellular target.

Accordingly, biological evaluation should distinguish toxicity or membrane effects caused by the free cyclodextrin from those of the vitamin–cyclodextrin system. Dose, cyclodextrin identity, substitution pattern, administration route, and duration of exposure should be reported explicitly. Such controls are essential before improved cellular uptake or permeability can be interpreted as a beneficial consequence of vitamin complexation.

Overall, the current literature provides considerably stronger evidence for cyclodextrin-dependent changes in vitamin stability, release, and local permeability than for altered transporter recognition, intracellular metabolism, systemic exposure, or physiological function. Establishing these later stages of the biological pathway represents one of the principal requirements for translating molecular cyclodextrin–vitamin interactions into biologically meaningful delivery strategies.

Interactions with biological macromolecules and broader physiological responses remain particularly understudied. Beyond vitamin-specific membrane transporters, there is little direct evidence showing whether cyclodextrin association alters subsequent interactions of water-soluble vitamins with binding proteins, enzymes, or other physiological macromolecules. Likewise, immunological consequences and effects on the intestinal microbiome have not been systematically evaluated for the vitamin–cyclodextrin systems identified in this review. These endpoints may become relevant particularly for repeated oral administration or for complex polymeric and nanoparticulate formulations, but the current evidence is insufficient to establish either beneficial or adverse effects. Future studies should therefore include these biological levels where appropriate rather than inferring them from physicochemical or permeability data.

### 6.6. Safety, Dose Dependence, and Translational Constraints of Cyclodextrins

The biological effects and translational suitability of cyclodextrins cannot be evaluated independently of the identity of the host, its degree and pattern of substitution, concentration, duration of exposure, and route of administration. Cyclodextrins should therefore not be considered a toxicologically uniform class. An excipient that is well tolerated after oral administration may not be suitable at comparable exposure by the parenteral route, while a derivative that efficiently modifies a biological membrane may simultaneously produce undesirable membrane disruption at higher concentrations.

A major mechanism underlying cyclodextrin-dependent biological effects is interaction with membrane lipids. β-Cyclodextrins can extract cholesterol and, depending on their structure and experimental conditions, other membrane components. This property is particularly pronounced for methylated β-cyclodextrins and explains both their usefulness as membrane-modifying agents and their greater potential for concentration-dependent cytotoxicity and hemolysis. Consequently, increased permeability observed in the presence of a cyclodextrin should not automatically be interpreted as evidence of improved physiological vitamin transport. The effect may instead result from nonspecific membrane perturbation, and the distinction becomes increasingly important as cyclodextrin concentration and exposure time increase.

Native cyclodextrins also differ substantially in their suitability for systemic administration. Native β-cyclodextrin has limited aqueous solubility and has historically been associated with renal toxicity after parenteral exposure, which strongly limits its use by this route. More hydrophilic derivatives, particularly hydroxypropyl-β-cyclodextrin (HPβCD) and sulfobutylether-β-cyclodextrin (SBEβCD), were developed in part to overcome these limitations and generally show more favorable parenteral compatibility. Nevertheless, improved compatibility does not imply absence of dose-dependent effects. Systemically administered cyclodextrins are substantially dependent on renal elimination, and accumulation may occur when renal function is impaired. Repeated high systemic exposure may therefore require particular caution [11,73].

The oral situation is different because systemic absorption of many cyclodextrins is low. Their safety profile is consequently generally more favorable by this route, although high oral doses may produce gastrointestinal effects. Thus, evidence obtained with an oral formulation cannot be transferred directly to parenteral, transdermal, ocular, nasal, or other routes. Local exposure may also be considerably higher than systemic exposure, particularly in formulations intentionally designed to alter epithelial permeability.

These considerations are directly relevant to several systems discussed in this review. For example, the increased transdermal transport of vitamin B12 produced by HPβCD-based polymeric penetration enhancers demonstrates a biologically meaningful permeability effect, but it should be interpreted in the context of cyclodextrin-induced modification of the skin barrier rather than as evidence that molecular B12 inclusion intrinsically improves absorption. Similarly, the use of concentrated cyclodextrin derivatives to facilitate corneal delivery of riboflavin requires distinction between reversible enhancement of epithelial permeability and nonspecific membrane perturbation. In both cases, appropriate controls with the free cyclodextrin or cyclodextrin-containing carrier are essential for assigning the observed biological effect.

From a translational perspective, four parameters should therefore accompany any claim of improved vitamin delivery: the exact cyclodextrin identity and substitution characteristics, its final concentration or administered dose, the duration and route of exposure, and evidence that the observed biological effect occurs within an acceptable safety range. The regulatory status and previous use of a particular cyclodextrin should also be considered route-specifically rather than generalized to the entire class. Importantly, regulatory acceptance of a cyclodextrin as an excipient for one route or product does not establish unrestricted safety for other routes, concentrations, or exposure durations.

Accordingly, the optimal cyclodextrin for a vitamin formulation should not be selected solely on the basis of the highest apparent association constant, loading efficiency, or permeability enhancement. Molecular recognition, formulation performance, membrane compatibility, systemic exposure, and route-specific safety must be evaluated together. This balance is particularly important for water-soluble vitamins, for which the benefit obtained from additional solubilization may be modest and therefore may not justify the use of a more membrane-active or less biocompatible cyclodextrin derivative.

### 6.7. Application Patterns

Pharmaceutical applications dominate for thiamine, pantothenate, B12, and vitamin C, particularly controlled release, topical delivery, and transdermal transport. Food applications are most prominent for vitamin C and riboflavin-related photochemistry, but direct addition of cyclodextrin to food does not consistently protect the vitamin and often changes aroma by binding unrelated volatile compounds. Cosmetic applications focus on topical vitamin C formulations and lipophilic ascorbate derivatives. Analytical applications are especially common for B6, vitamin C, thiamine, and folate, where cyclodextrin-modified electrodes, nanoclusters, quantum dots, and competitive displacement systems improve selectivity. These sensors demonstrate functional molecular recognition, but their complex interfacial behavior usually prevents direct extraction of a simple solution-phase inclusion constant.

Cyclodextrin-based formulations should also be considered in the context of alternative strategies for water-soluble vitamin delivery, including electrospun or spray-dried matrices, protein-based systems, liposomes, hydrogels, and polymeric nanoparticles [1,3,4,13]. A distinctive feature of cyclodextrins is their capacity for reversible molecular recognition, allowing them, in suitable systems, to modify the local environment of a vitamin without permanent covalent modification. This can be advantageous when the primary objective is protection of a labile molecular region, modulation of local concentration, or reversible control of release. However, cyclodextrins should not be regarded as universally superior carriers. Polymeric, liposomal, protein-based, and other encapsulation systems may provide effective protection or prolonged release through mechanisms that do not require molecular inclusion, and many cyclodextrin-containing formulations discussed in this review themselves rely substantially on such matrix-level effects. Direct head-to-head comparisons remain uncommon, making quantitative ranking of these approaches difficult. The most appropriate formulation should therefore be selected according to the principal limitation of the particular vitamin and route of administration rather than according to carrier class alone.

A separate group of studies uses vitamins in cyclodextrin-containing systems for purposes other than vitamin delivery. These include analytical sensors, photocatalytic materials, vitamin-functionalized targeting carriers, and multicomponent assemblies in which another compound is the actual cyclodextrin cavity guest. Although such studies are informative for understanding molecular recognition, competitive binding, covalent functionalization, or supramolecular organization, they should not be interpreted as evidence that cyclodextrins improve the stability, absorption, or bioavailability of the vitamin itself.

To avoid conflating these distinct applications with vitamin-delivery evidence, representative peripheral systems are summarized separately in Supplementary Table S1. They are retained because they clarify the actual molecular role of the vitamin and cyclodextrin, but they are excluded from the evidence synthesis concerning vitamin stabilization, release, permeability, pharmacokinetics, and translational performance.

### 6.8. Inclusion Versus Non-Inclusion Complexes: Terminological and Methodological Problems

#### 6.8.1. Terminological Categories

**Figure 3:**
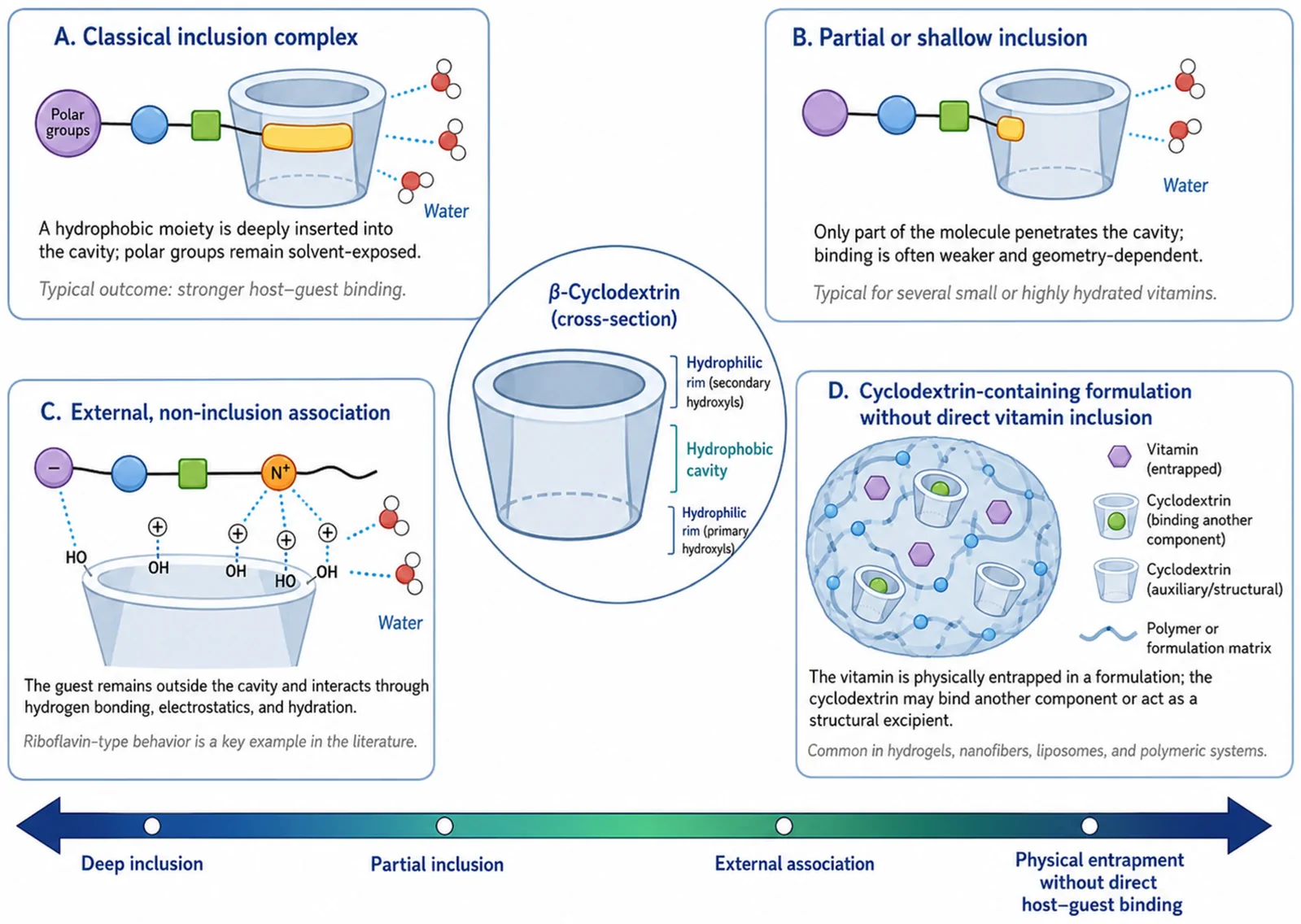
Possible modes of interaction between water-soluble vitamins and cyclodextrins.

The literature would benefit from a restricted and explicit vocabulary. A “binary inclusion complex” should denote a system in which a defined vitamin or vitamer penetrates the cavity of a defined cyclodextrin and the composition is supported by cavity-sensitive or spatial evidence. “Partial inclusion” should be used when only one molecular fragment enters the cavity. “External” or “non-inclusion association” should describe hydrogen bonding, electrostatic interaction, or hydrophobic contact at the rims or outer surface without cavity penetration. A “cyclodextrin-containing formulation” should be used when the vitamin is physically trapped in a matrix that contains cyclodextrin but direct host–guest binding has not been established. A “vitamin-functionalized cyclodextrin” is a covalent conjugate, as in biotin- or folate-appended hosts. Finally, a “cyclodextrin complex of a vitamin-containing conjugate” should identify cases such as biotin–BODIPY, in which the vitamin is only one part of a larger guest molecule (Figure 3).

These categories are not merely semantic. They determine what can be inferred about stoichiometry, reversibility, release, and mechanism. For example, B12-loaded αCD hydrogels release a vitamin from a poly(pseudo)rotaxane network, but the inclusion guest responsible for gelation is PEG, not B12 [56,57]. Biotinylated cyclodextrin dimers target cells through the covalently attached vitamin while complexing doxorubicin in their cavities [51]. Calling either system a B12–CD or biotin–CD inclusion complex obscures the actual molecular design.

#### 6.8.2. What Constitutes Evidence of Inclusion?

**Figure 4:**
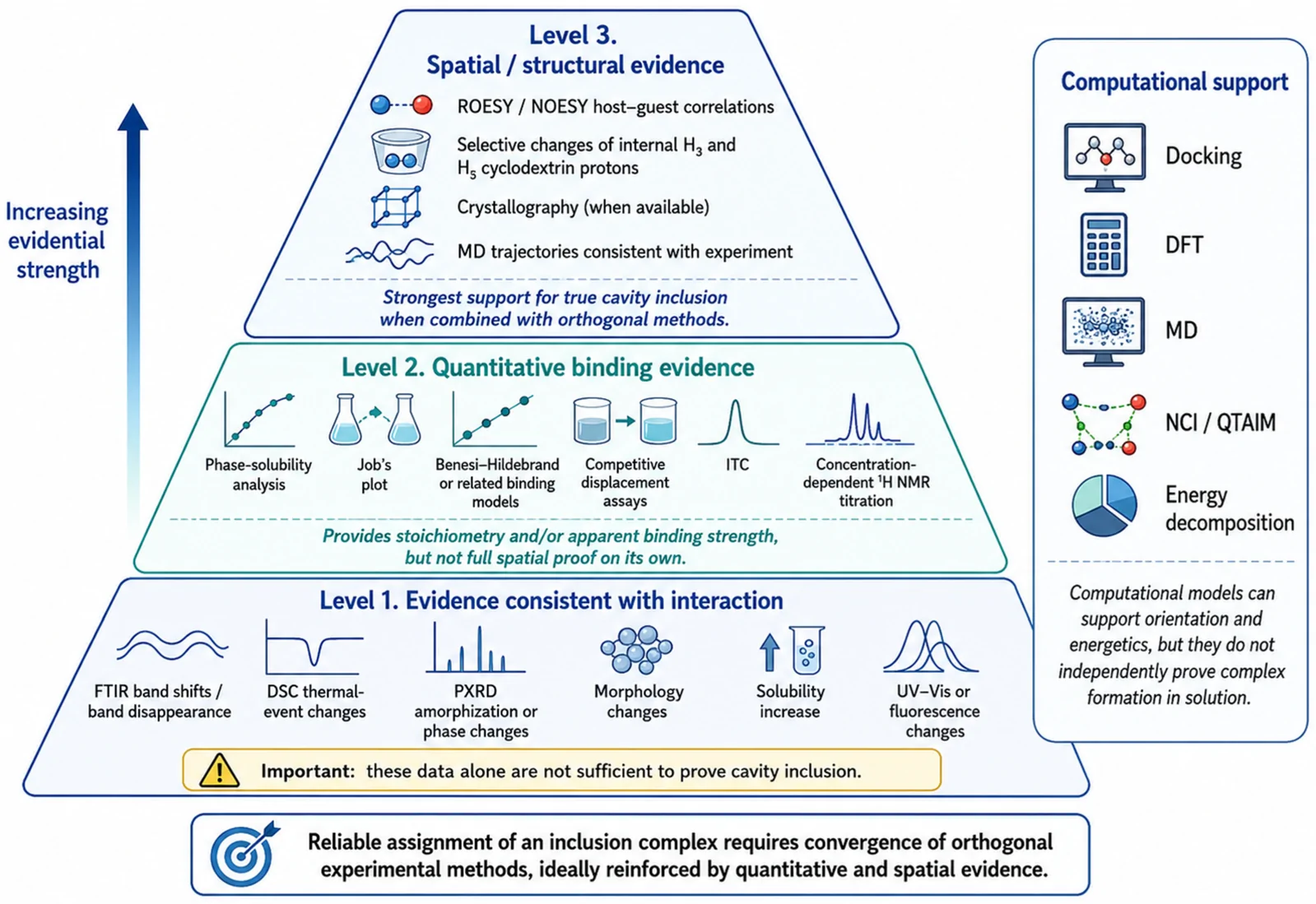
Hierarchy and complementarity of evidence used to identify cyclodextrin-water soluble vitamins inclusion complexes.

No single technique is universally decisive, but evidence can be ranked (Figure 4). Changes in FTIR bands, disappearance of a melting endotherm, loss of crystallinity in PXRD, or altered particle morphology demonstrate interaction and solid-state transformation. They do not locate the vitamin inside the cavity, because the same changes can result from hydrogen bonding, amorphous dispersion, salt formation, polymer entrapment, or dilution by the dominant cyclodextrin phase. Phase-solubility diagrams demonstrate cyclodextrin-dependent solubilization, but a linear A_L_ profile does not prove inclusion and can also reflect external complexes, aggregates, or micelle-like assemblies. Job plots provide an apparent dominant composition only under restrictive assumptions and are particularly vulnerable when the guest self-associates or when several complexes coexist.

One-dimensional NMR becomes more informative when changes in the inward-facing H3 and H5 protons are reported quantitatively and compared with external H1, H2, H4, and H6 shifts. ROESY or NOESY cross-peaks between vitamin protons and H3/H5 provide stronger evidence of spatial proximity, although exchange kinetics, concentration, and spectral overlap must be considered. DOSY can support association through a change in diffusion coefficient but may be insensitive to weak or rapidly exchanging complexes. The riboflavin case is exemplary: minimal H3/H5 changes, absent ROESY cross-peaks, nearly unchanged diffusion, and out-of-ring MD collectively support non-inclusion despite a positive phase-solubility slope [18].

ITC provides stoichiometry, affinity, and thermodynamics without requiring a chromophore, but weak heats, protonation reactions, buffer ionization, aggregation, and concentration errors can distort the fit. UV–visible and fluorescence methods are sensitive and convenient, yet changes in intensity can arise from polarity, aggregation, quenching, or instrumental inner-filter effects. Linearized Benesi–Hildebrand plots overweight low-concentration error and can yield apparently precise but biased constants; nonlinear fitting of raw data is preferable. Ideally, a solution constant should be confirmed by an orthogonal method and accompanied by residual analysis, uncertainty, and comparison of plausible stoichiometric models.

#### 6.8.3. Gas-Phase and Computational Evidence

ESI-MS is valuable for confirming that noncovalent host–guest ions can be generated and for identifying nominal composition, but ion abundance and collision stability do not directly report solution equilibrium. The folate study demonstrated this quantitatively: ESI-MS-derived affinities were approximately two orders of magnitude larger than the corresponding NMR/ITC values and suggested appreciable αCD association despite solution evidence favoring an exclusion complex [19]. Ion-mobility measurements and tandem mass spectrometry should therefore be interpreted primarily as gas-phase structural or stability information unless transfer from solution to the gas phase has been independently validated.

Computational approaches require similar caution. Docking, semiempirical calculations, DFT, and molecular dynamics can rationalize experimentally observed interactions and generate plausible binding geometries, but they do not independently establish the dominant solution-state structure. Their reliability depends on the treatment of protonation state, explicit or implicit solvation, relevant counterions, cyclodextrin substitution patterns, and conformational sampling. These factors are particularly important for water-soluble vitamins, whose charge state and hydration can change substantially under experimentally relevant conditions.

Optimizing one or a few preassembled host–guest geometries may identify local minima but does not establish their thermodynamic predominance. Likewise, docking scores and gas-phase interaction energies should not be interpreted as solution binding free energies. Stronger computational support is obtained when multiple starting orientations are examined, included and external binding modes are compared, explicit solvent and relevant ionic species are represented, and conformational sampling is sufficiently extensive to permit transitions between alternative states. Free-energy calculations or enhanced-sampling approaches can provide additional information, but their reliability remains dependent on the underlying molecular model and adequate sampling.

The nicotinic-acid studies illustrate the importance of molecular speciation: calculations performed for zwitterionic and neutral forms produced different preferred host–guest arrangements [37,38]. Neither optimized structure alone therefore establishes which geometry dominates under experimental solution conditions. By contrast, the riboflavin study used molecular dynamics together with one- and two-dimensional NMR, DOSY, and ROESY measurements, and the combined evidence supported predominantly out-of-cavity association [18]. Similarly, the recent vitamin C TIMS-MS/computational study [20] provides useful information on gas-phase ion structure and relative compactness, but it did not determine solution binding constants and therefore cannot establish the dominant equilibrium complex in solution.

Accordingly, computational results in this review are treated as supporting evidence only and are interpreted in the context of the corresponding experimental data. A single docking pose, optimized geometry, or gas-phase interaction energy is not considered sufficient evidence of molecular inclusion.

#### 6.8.4. Multicomponent Systems and Attribution of Effects

In multicomponent materials, each interaction must be assigned separately. A cyclodextrin may complex a hydrophobic co-active while the vitamin is bound to chitosan, trapped in PLGA, or dissolved in the aqueous phase. Curcumin/TPP–βCD plus chitosan/vitamin-C hydrogel systems and HPβCD–Peganum-alkaloid nanoparticles co-loaded with ascorbic acid are examples in which the vitamin is not the cyclodextrin guest [74,75]. Similarly, free vitamin C can act synergistically with vitamin-E–CD and cholesterol–CD in sperm cryopreservation without being complexed itself. Reporting should distinguish the cavity guest, matrix-bound component, free additive, covalent ligand, and analytical target.

A practical reporting standard would include: exact vitamin chemical form and purity; cyclodextrin type, degree of substitution, and molecular-weight distribution; host/guest feed ratio and independently measured stoichiometry; pH, buffer, ionic strength, temperature, and concentration range; preparation and purification details; comparison with physical mixture and empty carrier; at least one cavity-sensitive method for inclusion claims; quantitative fitting with uncertainty; mass balance or loading efficiency; stability-indicating assay for release studies; and controls that separate cyclodextrin effects from polymer, nanoparticle, and matrix effects. Adoption of these elements would make results across vitamins substantially more comparable.

## 7. Research Gaps and Future Perspectives

### 7.1. Understudied Vitamins and Vitamers

The evidence base identified in the present review is highly uneven, despite applying the same search strategy across all water-soluble vitamins considered. This unevenness represents a characteristic of the available literature rather than a difference in inclusion criteria. Vitamin C and riboflavin have comparatively extensive literature, whereas pantothenate, free biotin, and cobalamins remain poorly characterized at the molecular level. Future studies should examine whether small peripheral groups of cobalamins can form partial complexes, whether two or more cyclodextrins can bind cooperatively, or whether cyclodextrin polymers provide multivalent external binding. Such questions require solution NMR, calorimetry, scattering, and free-energy simulations rather than schematic depictions of complete encapsulation.

**Figure 5:**
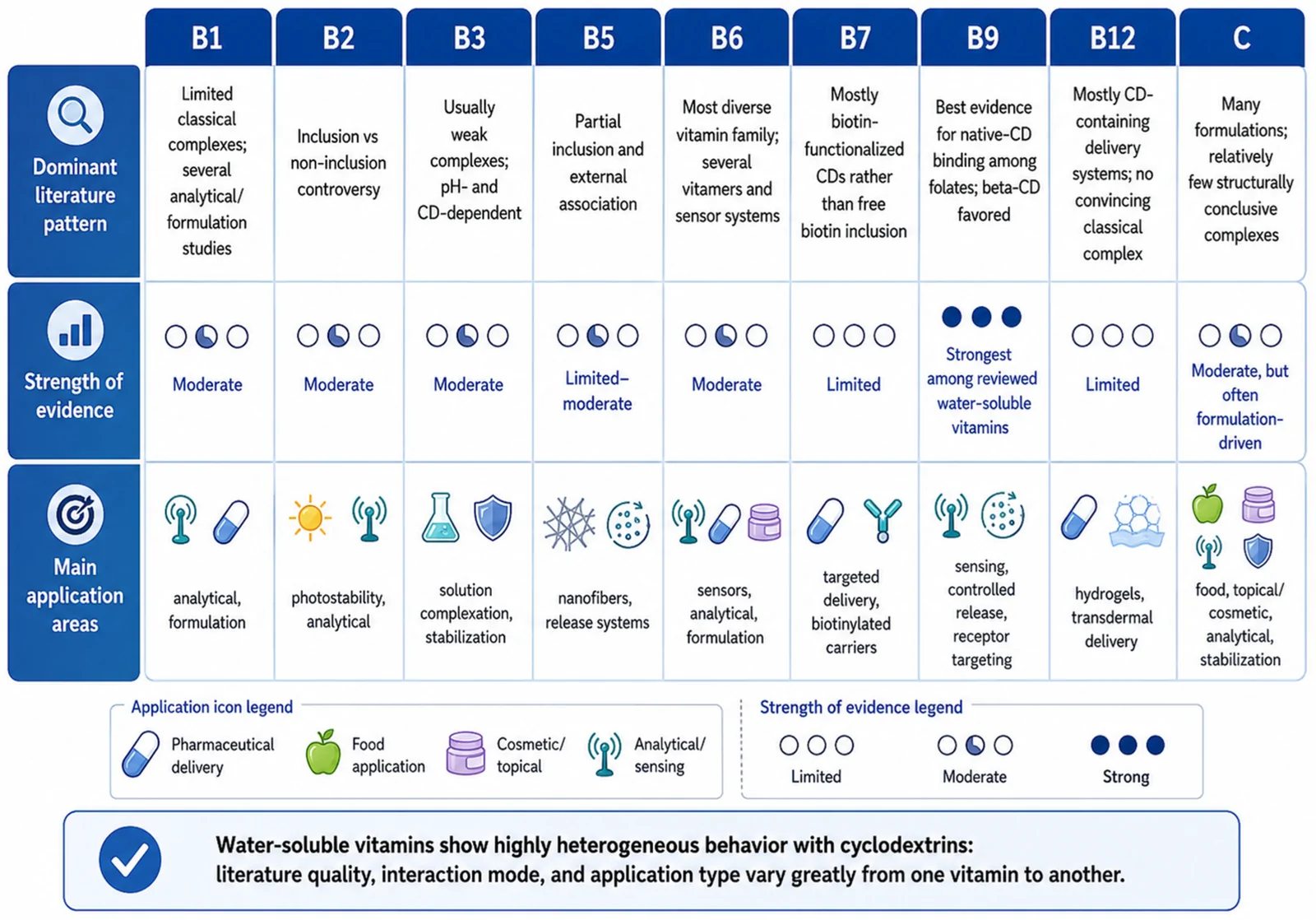
Comparative landscape of cyclodextrin interactions with water-soluble vitamins and the current strength of evidence linking molecular association to functional or biological outcomes. Strong: Grade A molecular evidence and/or directly demonstrated functional or biological effects attributable to the characterized vitamin–cyclodextrin system; Moderate: predominantly Grade B molecular evidence or functional effects with only partial mechanistic attribution; Limited: predominantly Grade C, indirect, formulation-level, or sparse evidence without a demonstrated causal molecular-to-biological link. Ratings are based on the studies summarized in Table 2 and Table 3 and the corresponding vitamin-specific sections.

Important vitamers are also missing (Figure 5). Pyridoxamine and pyridoxamine-5′-phosphate have received little attention compared with pyridoxine, pyridoxal, and PLP. Pantothenic-acid studies should distinguish free acid, calcium D-pantothenate, and hemicalcium DL salts. Riboflavin research should separate the parent vitamin from alloxazine, lumichrome, and other photoproducts. Folate studies should compare sodium folate with physiologically relevant reduced folates, whose charge and conformational flexibility differ substantially. These comparisons may reveal that vitamer identity is a stronger determinant of complexation than the nominal vitamin label.

The qualitative evidence categories summarized in Figure 5 were assigned using the evidence-assessment framework defined in Section 2.2. and the studies summarized in Table 2 and Table 3. Peripheral systems summarized separately in Supplementary Table S1 were not considered evidence for improved vitamin stabilization, delivery, or bioavailability unless the vitamin itself was the directly studied cyclodextrin-associated cargo.

### 7.2. Solution Speciation, Aggregation, and Standardized Thermodynamics

pH-dependent speciation and self-association are major unresolved variables. Nicotinic acid, folic acid, ascorbic acid, PLP, and pantothenate can change charge state within experimentally relevant pH ranges, while folate and riboflavin can self-associate. Future studies should use independently measured pH, appropriate activity or speciation calculations, and global models that include protonation and guest aggregation. Counterions and buffer enthalpy should be reported, especially for ITC. Where binding is weak, high-sensitivity NMR and competitive methods should be combined with direct calorimetry only when the heat signal is sufficient.

A multicenter or benchmark study using a small set of vitamins and standardized conditions would be particularly valuable. The same host–guest pairs could be measured by nonlinear UV/fluorescence fitting, NMR titration, ITC, and phase solubility at defined temperature, pH, and ionic strength. Such a study would quantify method-dependent bias and establish reference constants. The thiamine, nicotinic-acid, riboflavin, and folate discrepancies show that the field currently lacks this calibration.

Competitive binding in multivitamin formulations represents an additional largely unexplored problem. Because food and pharmaceutical products frequently contain several vitamins simultaneously, association constants measured for isolated binary systems may not predict cyclodextrin occupancy in the final formulation. Direct competition experiments using the same cyclodextrin, identical solution conditions, and defined mixtures of vitamers would be required to establish true selectivity and to determine whether preferential binding or displacement alters the free-vitamin fraction. The currently available heterogeneous binary data are insufficient for a reliable quantitative selectivity ranking.

### 7.3. From Structural Characterization to Translational Performance

The principal biological limitation of the current literature is the incomplete connection between molecular cyclodextrin–vitamin interactions and experimentally demonstrated changes in biological exposure or response.

A rigorous biological-validation pathway should proceed through several experimentally distinct levels. First, the cyclodextrin-containing formulation should be compared with the free vitamin, free cyclodextrin, physical mixture, and empty carrier under exposure-matched conditions, while confirming by a stability-indicating assay that the measured material represents chemically intact vitamin. Second, barrier models should distinguish increased apparent permeability from true cellular uptake and, where physiological transporters are involved, transporter-specific studies should determine whether cyclodextrin association promotes delivery to the membrane, inhibits recognition, or requires prior dissociation. Third, cellular studies should quantify intracellular vitamin concentrations and, where relevant, formation of active coenzyme or metabolite forms rather than relying solely on nonspecific viability or functional assays. Fourth, route-appropriate animal pharmacokinetic studies should directly compare the cyclodextrin formulation with the corresponding free vitamin and determine systemic or local exposure, including concentration–time profiles and, when relevant, tissue distribution or retention. Because endogenous vitamin pools can complicate interpretation, isotope-labeled or otherwise analytically distinguishable tracers may be particularly valuable. Finally, any pharmacokinetic advantage should be linked to a vitamin-specific physiological or pharmacodynamic endpoint and evaluated together with cyclodextrin dose, safety, and tolerability before clinically meaningful superiority over a conventional formulation can be claimed.

Many publications report enhanced “bioavailability” without pharmacokinetic data. The next generation of studies should connect molecular association to measurable absorption, tissue exposure, or local retention. For oral systems, dissolution and simulated digestion should be followed by permeability and in vivo pharmacokinetics. For topical vitamin C, stability-indicating quantification should accompany skin-deposition and permeation studies. For B12 penetration enhancers, it would be useful to compare flux with polymer architecture, free HPβCD, and barrier-recovery kinetics. For food systems, isotope- or chromatographic tracking could determine whether protection results from direct vitamin binding or from sequestration of other matrix components.

Release studies also require improved design. “Cumulative release” cannot decrease; a declining measured concentration indicates degradation, adsorption, or analytical loss. Vitamin C and riboflavin studies should therefore distinguish released mass from chemically intact mass using stability-indicating HPLC or LC-MS. Sink conditions, membrane effects, carrier erosion, and total mass recovery should be reported. Mathematical models should not be used solely on the basis of the highest R^2^; mechanism-based selection and residual analysis are needed.

### 7.4. Advanced Cyclodextrin Architectures

**Figure 6:**
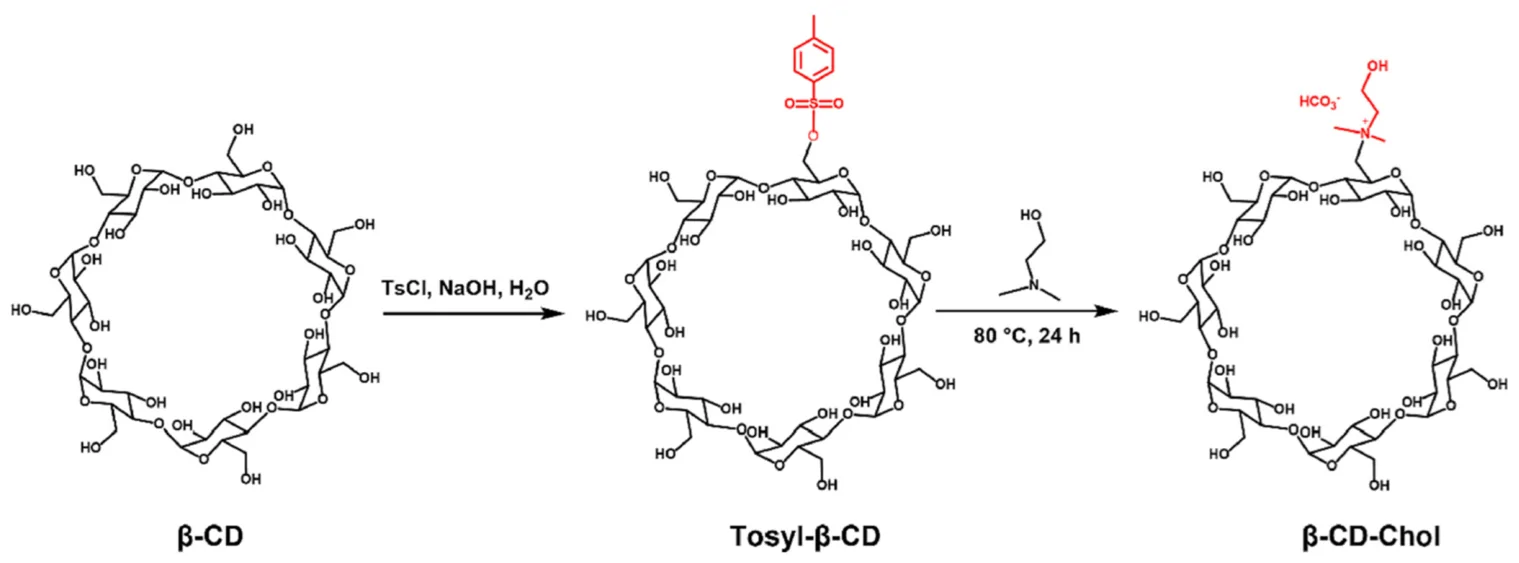
Illustrative example of cyclodextrin functionalization: synthetic route for the preparation of a mono-substituted cationic β-cyclodextrin bearing a choline-like pendant (β-CD-Chol). Reproduced from [76] under CC BY-NC-ND 4.0. The scheme illustrates one strategy for introducing additional functionality into the cyclodextrin scaffold and is not intended to represent the full range of advanced cyclodextrin architectures.

Native αCD, βCD, and γCD remain useful mechanistic hosts, but tailored derivatives may provide better selectivity for highly polar vitamins. Systematic libraries varying charge, substitution pattern, spacer length, and cavity multiplicity could be screened against defined vitamers. Sulfobutylether and cationic derivatives may strengthen ionic association, while dimers and polymers may bind large or multivalent guests such as folates and cobalamins. However, increasing architectural complexity also increases the number of external binding sites, making rigorous controls even more important (Figure 6).

One illustrative strategy for expanding cyclodextrin functionality is the covalent introduction of charged pendant groups. Figure 6 shows the synthesis of a mono-substituted cationic β-cyclodextrin bearing a choline-like pendant [76]. Although this particular derivative was not developed for vitamin delivery, it illustrates how the native cyclodextrin scaffold can be chemically modified to introduce additional electrostatic and biological functionality while retaining the cyclodextrin framework. Such approaches may be relevant to future water-soluble vitamin systems, for which charge, hydration, and external interactions can be as important as cavity fit. Figure 6 should therefore be regarded as an illustrative example of cyclodextrin functionalization rather than as a representative synthetic route for all advanced cyclodextrin architectures.

Stimuli-responsive materials are a promising direction when their design is based on a known molecular interaction. Redox- or enzyme-cleavable cyclodextrin polymers, competitive displacement systems, and photoresponsive carriers could protect vitamins during processing and release them at a desired site. The analytical B6 systems show that competitive binding can be highly sensitive; analogous principles could be adapted to controlled delivery, provided that the relative solution affinities are measured by comparable methods rather than inferred from gas-phase or unrelated data.

### 7.5. Implications of the Evidence Framework for Future Studies

Application of the evidence framework used in this review highlights a substantial gap between formulation-level evidence and rigorous demonstration of molecular inclusion. Grade A should be regarded as requiring convergent quantitative and cavity-specific solution evidence, whereas Grade B identifies systems for which association is supported but the detailed molecular geometry remains incompletely established. Grade C identifies systems in which interaction is inferred predominantly from indirect, solid-state, computational, gas-phase, or formulation-level observations. Importantly, these grades describe the strength of molecular evidence rather than the technological or biological value of a formulation; a Grade C system may still exhibit substantial biological or pharmaceutical utility.

Future studies can move systems toward stronger molecular characterization by combining quantitative solution thermodynamics with cavity-sensitive NMR or equivalent spatial methods and by explicitly evaluating competing external and inclusion geometries. Conversely, biological and translational evidence should be assessed independently, because demonstrating molecular inclusion does not by itself establish improved absorption, cellular exposure, pharmacokinetics, or biological efficacy. Maintaining these two evidence dimensions separately should make future comparisons between cyclodextrin–vitamin systems more rigorous and biologically meaningful.

## 8. Conclusions

The available evidence demonstrates that interactions between cyclodextrins and water-soluble vitamins are highly system-dependent and cannot be described by a universal inclusion model. The most convincing molecular evidence is available for thiamine with native α- and β-cyclodextrins [22], nicotinic acid with native cyclodextrins [34,36], sodium folate with β- and γ-cyclodextrin [19], and L-ascorbic acid with β-cyclodextrin [36]. These systems are supported by complementary quantitative solution measurements together with NMR, ROESY, ITC, or other structurally informative methods. Importantly, strong molecular evidence does not necessarily indicate classical cavity inclusion: for riboflavin, complementary NMR, DOSY, ROESY, and molecular-dynamics results instead support predominantly external or non-inclusion association under the investigated conditions [18]. Evidence for pantothenate and several vitamin B6 systems is less definitive, while convincing binary inclusion of free biotin or cobalamins has not been demonstrated in the literature reviewed here.

Across the vitamin series, the most consistently demonstrated functional benefits of cyclodextrin-containing systems are improved chemical or photochemical stability, modified release, and, in selected formulations, enhanced local permeability. These outcomes cannot automatically be attributed to molecular inclusion because polymer organization, physical entrapment, membrane perturbation, and interactions with other formulation components frequently contribute substantially. In particular, vitamin-functionalized carriers, analytical systems, and multicomponent formulations in which another molecule is the actual cyclodextrin guest should not be considered evidence for stabilization or enhanced bioavailability of the free vitamin.

The principal translational gap is therefore not the absence of cyclodextrin-containing vitamin formulations, but the limited demonstration of a causal progression from molecular association to improved biological exposure. Convincing evidence for enhanced systemic vitamin bioavailability attributable specifically to cyclodextrin complexation remains scarce, and clinically validated benefits have not been established for the systems considered in this review. Future studies should therefore combine rigorous solution-state characterization with appropriate formulation controls, permeability and cellular experiments, and, where relevant, pharmacokinetic and in vivo assessment. Such an integrated approach is necessary to determine when cyclodextrin–vitamin interactions represent a biologically meaningful delivery strategy rather than only an interesting supramolecular or formulation phenomenon.

## Figures and Tables

**Table 1:** Comparison of representative previous reviews with the scope of the present review.

Review	Main Scope	Vitamins Covered	Treatment of Molecular Evidence	Biological/Translational Endpoints	Principal Distinction
Coelho et al., 2022 [1]	Delivery of water-soluble vitamins by electrospinning and spray drying	Water-soluble vitamins	Limited; not focused on CD host–guest interactions	Stability, encapsulation, delivery, food/nutraceutical applications	Technology- and processing-oriented review
Kali et al., 2024 [6]	Cyclodextrins and derivatives in advanced drug delivery	Not vitamin-specific	General host–guest and formulation perspective	Permeation, cellular uptake, clinical trials, advanced products	Broad pharmaceutical CD-delivery review
Saffarionpour and Diosady, 2025 [12]	Cyclodextrins for micronutrient delivery	Vitamins together with iron and iodine	Inclusion complexes and CD-containing delivery systems	Stability, controlled release, permeation, food/pharmaceutical/cosmeceutical applications	Broad micronutrient-oriented CD review
Zielińska-Pisklak et al., 2025 [14]	Cyclodextrin complexes of fat-soluble vitamins	Vitamins A, D, E, and K	Detailed physicochemical and structural characterization	Solubilization, absorption enhancement, pharmaceutical applications	Dedicated review of fat-soluble vitamin–CD complexes
Deng et al., 2026 [13]	Encapsulation of water-soluble vitamins for food fortification	Mainly folate (B9) and vitamin C	Delivery-system characterization; not CD-specific	Gastrointestinal stability, absorption, metabolism, nutritional efficacy	Food-fortification and nutritional-delivery perspective
Kumar et al., 2026 [11]	Cyclodextrin synthesis, pharmaceutical applications, drug delivery, and safety	Not vitamin-specific	Broad structural and analytical discussion	Pharmacokinetics, toxicity, safety, regulatory considerations	Comprehensive pharmaceutical and safety perspective
Present review	Cyclodextrin interactions with water-soluble vitamins from molecular association to biological relevance	B1, B2, B3, B5, B6, B7, B9, B12, and C	Explicit distinction between inclusion, partial inclusion, external association, entrapment, and covalent functionalization; graded evaluation of evidence	Stability, release, permeability/uptake, biological effects, in vivo evidence, and translational limitations	Direct integration of the strength of molecular evidence with functional and biological consequences

**Table 2:** Representative and best-characterized molecular interactions between water-soluble vitamins and cyclodextrins.

Vitamin/Vitamer	Cyclodextrin	Preparation/Medium	Stoichiometry	Association or Stability Constant	Principal Evidence and Proposed Mode	Evidence Grade	Ref.
B1, thiamine HCl	αCD	Aqueous mixing, cooling, filtration and drying	1:1	1.12–1.28 × 10^3^ M^−1^ at 298 K (UV bands/fits)	Job plot, UV–Vis, ESI-MS, ^1^H NMR and ROESY; cavity participation, dynamic orientation	A	[22]
B1, thiamine HCl	βCD	Aqueous mixing, cooling, filtration and drying	1:1	1.67–1.90 × 10^3^ M^−1^ at 298 K	Job plot, UV–Vis, ESI-MS, ^1^H NMR and ROESY; stronger binding than αCD	A	[22]
B2, riboflavin	βCD	Phase-solubility and dilute-solution spectroscopy/NMR	Nominal 1:1 model	39.7 ± 0.31 M^−1^	No cavity ROESY correlations; DOSY and MD favor out-of-ring hydrogen bonding	A (non-inclusion)	[18]
B2, riboflavin	HPβCD	Phase-solubility and dilute-solution spectroscopy/NMR	Nominal 1:1 model	20.6 ± 0.22 M^−1^	No cavity ROESY correlations; external-rim association in MD	A (non-inclusion)	[18]
B3, nicotinic acid (zwitterion)	αCD	Aqueous NMR and calorimetry, pH 3.3–3.5	1:1	23.4 ± 0.8 kg mol^−1^ (NMR); 33 ± 5 kg mol^−1^ (calorimetry)	Shallow, tight inclusion; enthalpy-driven with unfavorable entropy	B	[34]
B3, nicotinic acid (zwitterion)	βCD	Aqueous NMR and calorimetry, pH 3.3–3.5	1:1	6.5 ± 0.5 kg mol^−1^	Deeper inclusion but poorer fit; dehydration gives favorable entropy contribution	B	[34]
B3, nicotinic acid	βCD	Job plot, UV–Vis, ROESY and ITC	1:1	1.23 × 10^3^ M^−1^ (nonlinear UV); 1.498 ± 0.155 × 10^3^ M^−1^ (ITC)	Pyridine ring in cavity; enthalpy-driven binding	A	[36]
B5, calcium D-pantothenate	βCD and HPβCD	Aqueous 1:1 mixtures; volumetric, acoustic and NMR measurements	Not independently determined	Not reported	H3/H5 shifts plus external interactions; βCD stronger; partial inclusion/external association	B	[42]
B6, PLP	βCD	Kneading equimolar components	1:1	5772.3 M^−1^	Fluorescence, ^1^H NMR, FTIR, LC-MS and docking; heteroaromatic region cavity-bound	B	[45]
B9, sodium folate	αCD	D2O NMR titration; ESI-MS	Approximately 1:1	<5 M^−1^ by NMR	Largest host shift at external H6; exclusion rather than cavity inclusion	A (exclusion)	[19]
B9, sodium folate	βCD	D2O NMR, water/HEPES ITC and ESI-MS	1:1	118 ± 11 M^−1^ (NMR); 162 ± 13 M^−1^ (ITC water); 136 ± 11 M^−1^ (HEPES)	H3/H5 and aromatic shifts; strongest native-CD solution complex	A	[19]
B9, sodium folate	γCD	D2O NMR and ITC	1:1	17 ± 2 M^−1^ (NMR); 56 ± 26 M^−1^ (ITC)	Cavity participation but loose fit and weaker binding than βCD	A	[19]
C, sodium ascorbate	αCD	Equimolar aqueous solution, nitrogen, freeze-drying	1:1 model	112 M^−1^ (kinetically derived apparent constant)	Saturable photodegradation protection and DSC; no cavity-specific NMR	B	[60]
C, L-ascorbic acid	βCD	Job plot, UV–Vis, surface tension, conductivity, ROESY and ITC	1:1	3.06 × 10^3^ M^−1^ (nonlinear UV); 3.655 ± 0.335 × 10^3^ M^−1^ (ITC)	ROESY correlations with H3/H5; enthalpy-driven inclusion	A	[36]
C, L-ascorbic acid	αCD, βCD and γCD	Aqueous 1:1 mixtures; TIMS-TOF-MS and calculations	Dominant 1:1 ions	No solution constants	βCD most compact calculated fit; γCD most collision-resistant gas-phase ion	C (gas phase)	[20]

Evidence grade A: quantitative solution analysis plus cavity-specific evidence (typically ROESY/NOESY or equivalent). Grade B: several complementary techniques, but no decisive spatial evidence or no independently established stoichiometry. Grade C: mainly solid-state, indirect, computational, or formulation-level evidence. Constants are reported in the units used by the original authors; values expressed in kg mol^−1^ were obtained on a molality scale and should not be compared directly with M^−1^ values.

**Table 3:** Cyclodextrin-containing systems relevant to the stabilization, release, permeability, or delivery of water-soluble vitamins in which direct molecular inclusion is partial, indirect, or unproven.

Vitamin	System	Actual Role of Cyclodextrin	Actual Role of Vitamin	Functional/Biological Outcome	Recommended Terminology	Ref.
B1	Chitosan–gelatin hydrogel containing βCD–thiamine complex	Host and component of hydrogel-loaded complex	Cavity guest/released nutrient	Slower release than directly loaded thiamine; biodegradable carrier	βCD–thiamine complex incorporated into hydrogel	[23]
B1	βCD wall material in microcapsules	Wall/matrix component	Encapsulated nutrient	Improved stability in lipid-rich systems	Thiamine-containing βCD microcapsules; inclusion not independently established	[24]
B2	γCD plus multicomponent liposomes	Proposed riboflavin host within liposomal aqueous phase	Photosensitive cargo	Photoprotection balanced against entrapment efficiency	γCD/riboflavin-containing protective liposomes	[30]
B2	Cationic poly(βCD-co-guanidine)	Multiple cavity and external/electrostatic binding sites	Partly cavity-associated and partly network-bound cargo	pH-dependent biphasic release	Polymeric βCD carrier with inclusion and non-inclusion binding	[28]
B2	Peracetylated βCD polymer nanofibers	Hydrophobic polymeric carrier	Entrapped/associated cargo	Very prolonged release	Riboflavin-loaded βCD-polymer nanofibers	[29]
B2 derivative	Lumichrome/HPβCD collagen hydrogel	Solubilizing host and hydrogel-associated complex	Photoreactive guest	Photocrosslinking, fibroblast studies and antibacterial photochemistry	Lumichrome–HPβCD complex in collagen hydrogel	[32,33]
B3	HPβCD/Eudragit S100 nanoemulsion	Polymeric formulation component and possible weak host	Loaded nicotinamide	Sustained antimicrobial release	HPβCD-containing nicotinamide delivery system	[39]
B5	HPβCD/pantothenate electrospun nanofibers	Host and fiber-forming excipient	Associated pantothenate salt	Antibacterial activity and altered thermal behavior	Pantothenate–HPβCD supramolecular nanofibers	[41]
B9	βCD-containing chitosan/collagen scaffold	Matrix additive and presumed folate host	Loaded folic acid	Prolonged release and neuronal-cell compatibility	Folic-acid-loaded βCD-containing scaffold	[55]
B12	αCD/PLGA–PEG–PLGA hydrogel	Threads PEG blocks to form supramolecular network	Physically trapped hydrophilic cargo	Sustained release over weeks	Vitamin-B12-loaded αCD/polymer hydrogel	[56]
B12	αCD/PCL–PEG–PCL hydrogel	Threads PEG blocks; network crosslinker	Physically trapped cargo	Sustained release; internally inconsistent endpoint reporting	Vitamin-B12-loaded αCD/polymer hydrogel	[57]
B12	HPβCD–PEI transdermal enhancer	Polymeric penetration enhancer acting on skin barrier	Model hydrophilic permeant	Approximately sixfold enhancement of transport	HPβCD-based penetration enhancer for B12	[58]
B12	HPβCD–PEI–MPC transdermal enhancer	Zwitterionic penetration enhancer	Model hydrophilic permeant	Approximately 20–40-fold higher flux	Zwitterionic HPβCD-based penetration enhancer for B12	[59]
C	αCD–sodium-ascorbate in liposomes with light absorbers	Host plus component of multibarrier formulation	Photosensitive guest	Up to ~120-fold photostabilization only for full combination	Sodium-ascorbate–αCD complex in protective liposomes	[60]
C	βCD/ascorbic-acid cellulose-acetate/PVA topical material	Presumed host immobilized on carrier	Released active	Near-zero-order topical release	βCD–ascorbic-acid-loaded nanofiber patch	[63]
C	SBEβCD-crosslinked chitosan nanoparticles	Ionic crosslinker and possible host	Loaded vitamin	~78% association and multiday release	Vitamin-C-loaded SBEβCD/chitosan nanoparticles	[66]
C derivative	Ascorbyl tetraisopalmitate/γCD	Host for hydrophobic chains, probably multivalent/partial	Lipophilic derivative guest	One-year content stability and enhanced release in ethanol	γCD complex of a lipophilic vitamin-C derivative	[67]
C	βCD or HPβCD added directly to juices/foods	Matrix modifier; binds multiple food components	Food nutrient/quality marker	No effect in some juices; modest matrix-dependent protection in others	Cyclodextrin-treated food matrix, not a binary complex	[68,69,70]

**Table 4:** Cross-study evidence linking cyclodextrin–vitamin association with functional, biological, and translational outcomes.

Vitamin/Representative System	Molecular Association	Stability	Release	Permeability	Cellular/Biological Effect	Animal PK	Clinical Outcome	Ref.
B1, thiamine–αCD/βCD	Yes, directly characterized	Limited	Transfer/release demonstrated	—	—	—	—	[22]
B1, βCD–thiamine in chitosan–gelatin hydrogel	Supported	—	Improved/controlled	—	—	—	—	[23]
B2, riboflavin–βCD/HPβCD	Yes, predominantly non-inclusion under the studied conditions	—	—	—	Cellular effects reported	—	—	[18]
B2, riboflavin with CD-based polymeric carriers	Partial/mixed or formulation-level	—	Prolonged/controlled	—	—	—	—	[28,29]
B2, riboflavin with CD derivatives for corneal delivery	Association present; contribution of cavity inclusion uncertain	—	—	Enhanced corneal permeability	—	—	—	[21]
B3, nicotinic acid–αCD/βCD	Yes, quantitatively characterized	—	—	—	—	—	—	[34,35,36]
B5, pantothenate–HPβCD nanofibers	Supported, but cavity geometry incompletely established	Improved thermal/formulation behavior	—	—	Antibacterial and cellular effects evaluated	—	—	[41]
B6, PLP–βCD	Yes, quantitatively supported	—	—	—	No vitamin-delivery outcome demonstrated	—	—	[45]
B9, sodium folate–βCD	Yes, quantitatively characterized	—	—	—	—	—	—	[19]
B9, folic-acid-loaded βCD/chitosan/collagen scaffold	Inclusion unproven	Improved formulation behavior	Prolonged	—	Cellular compatibility demonstrated	—	—	[55]
B12, αCD/polymer supramolecular hydrogels	No direct B12 cavity inclusion; physical entrapment	—	Prolonged	—	—	—	—	[56,57]
B12, HPβCD-based polymeric penetration enhancers	No direct B12 inclusion demonstrated	—	—	Strongly enhanced transdermal transport	—	No vitamin PK demonstrated	—	[58,59]
C, sodium ascorbate–αCD in protective liposomes	Molecular association supported	Strong photoprotection in the complete formulation	—	—	—	—	—	[60]
C, L-ascorbic acid–βCD	Yes, quantitatively and spatially supported	—	—	—	—	—	—	[36]
C, βCD/ascorbic-acid topical material	Presumed/supported indirectly	—	Near-zero-order release	—	—	—	—	[63]
C, SBEβCD/chitosan nanoparticles	Association indirect within multicomponent system	—	Multiday release	—	—	—	—	[66]
C derivative, ascorbyl tetraisopalmitate–γCD	Partial/multivalent association probable	Long-term content stability	Enhanced release	—	—	—	—	[67]

Interpretation: “Yes” indicates direct experimental evidence for the stated endpoint; “supported” indicates evidence that is substantial but does not fully establish the proposed molecular mechanism; “—“ indicates that the endpoint was not demonstrated in the reviewed study or studies. Formulation-level effects were not attributed to molecular inclusion unless the corresponding host–guest interaction had been independently demonstrated.

## Data Availability

The original contributions presented in this study are included in the article. Further inquiries can be directed to the corresponding author.

## Supplementary Materials

The following supporting information can be downloaded at https://www.mdpi.com/article/10.3390/biology15181637/s1, Table S1. Peripheral cyclodextrin–vitamin systems involving analytical sensing, vitamin-functionalized carriers, photocatalytic applications, or multicomponent assemblies in which the vitamin is not the principal cyclodextrin cavity guest. Table S2. Additional molecularly characterized cyclodextrin interactions with water-soluble vitamins not retained in the condensed Table 2 of the main manuscript.
