# Supplementary material for: Cyclodextrin Interactions with Water-Soluble Vitamins: Biological Relevance, Stability, Delivery, and Translational Implications—A Critical Review
Source: Biology (Basel). 2026 Sep 16;15(18):1637. doi: 10.3390/biology15181637 (PMC13604713; doi:10.3390/biology15181637)
Supplement: Supplementary file 1 [file biology-15-01637-s001.zip › biology-4559427-supplementary.pdf]

Table S1. Peripheral cyclodextrin–vitamin systems involving analytical sensing, vitamin-functionalized carriers, photocatalytic applications, or multicomponent assemblies in which the vitamin is not the principal cyclodextrin cavity guest.

Vitamin	System	Actual role of cyclodextrin	Actual role of vitamin	Functional / biological outcome	Recommended terminology	Ref.
B1	βCD-based potentiometric membrane	Ionophore / recognition element	Analyte	Determination in multivitamin formulations	Cyclodextrin-based sensor for thiamine	[25]
B2	Furaneol–βCD photochemical system	Host of furaneol; external organizer for riboflavin	External photosensitizer	Accelerated sensitized oxidation	Non-inclusion riboflavin association with a substrate–βCD complex	[31]
B3	ZnO/βCD/nicotinic-acid nanocomposite	Component of inorganic hybrid	Surface-/matrix-associated nicotinic acid	Photocatalytic, antimicrobial and cytotoxic tests	Multicomponent ZnO/βCD/nicotinic-acid material	[40]
B6	βCD-stabilized Ag nanoparticles bearing PLP	Surface stabilizer and PLP host	Complexed cofactor / recognition unit	Hydrazine fluorescence sensing	PLP–βCD complex displayed on Ag nanoparticles	[45]
B6	βCD–Cu nanoclusters bearing pyridoxal	Surface host / fluorophore environment	Displaceable B6 cofactor	Folic-acid sensing	Pyridoxal–βCD sensing assembly	[46]
B6	βCD–ZnO quantum dots with PLP or pyridoxal	Nanoparticle surface modifier and presumed host	Recognition cofactor	Histamine sensing	βCD-functionalized nanoprobes containing B6 vitamers	[47]
B6	βCD/N-doped graphene quantum dots with pyridoxal	Surface host / photophysical modifier	Recognition cofactor	Dopamine sensing	βCD-containing pyridoxal nanoprobe	[48]
B7	Biotin-functionalized CD dimers with doxorubicin	Covalently biotinylated host; doxorubicin cavities	Targeting ligand, not guest	Transporter-targeted doxorubicin delivery	Biotin-functionalized cyclodextrin dimer	[51]

B7	Biotinylated HPβCD redox-responsive micelles	Covalently modified polymeric carrier	Targeting ligand	Paclitaxel delivery and imaging	Biotin-functionalized HPβCD micelles	[52]
B7	Biotinylated βCD-coated Bi2O3 nanoparticles	Surface coating functionalized with biotin	Covalently attached ligand	Antimicrobial and anticancer testing	Biotinylated βCD-coated nanoparticles	[53]
B7	Biotin–BODIPY/cationic βCD nanosheets	Host for a large hydrophobic conjugate	Part of the guest and targeting motif	Combined photodynamic/chemodynamic therapy	βCD complex of a biotin-containing BODIPY conjugate	[54]
B9	βCD–Cu nanocluster/pyridoxal displacement sensor	Host for pyridoxal and nanocluster modifier	Competing analyte	Folic-acid detection	Cyclodextrin-based displacement assay for folate	[46]
C	HPβCD in capillary electrophoresis	Transient selector affecting mobility	Analyte	Improved separation from preservatives	HPβCD-assisted electrophoretic determination of ascorbate	[71]
C	βCD/CNT electrochemical electrode	Surface recognition and catalytic interface	Analyte	LOD 0.21 μM; simultaneous drug/vitamin analysis	βCD/CNT sensor with proposed vitamin-C host–guest interaction	[72]

Table S2. Additional molecularly characterized cyclodextrin interactions with water-soluble vitamins not retained in the condensed Table 2 of the main manuscript.

Vitamin / vitamer	Cyclodextrin	Preparation / medium	Stoichiometry	Association or stability constant	Principal evidence and proposed mode	Evidence grade	Ref.
B1, thiamine HCl	βCD	Microwave-assisted preparation; solid isolated and loaded into hydrogel	1:1	122.437 ± 4 M^{-1}	A_L phase solubility, FTIR, PXRD, DSC, microscopy, docking; proposed inclusion	B	[23]
B2, riboflavin	αCD	Aqueous solubility and NMR titration	1:1	3.9 ± 0.2 kg mol^{-1} (solubility); 6 ± 1 kg mol^{-1} (NMR)	Very weak, shallow association; methyl/ring region near cavity	B	[26]

B2, riboflavin	βCD	Aqueous solubility and NMR titration	1:1	38 ± 2 kg mol^{-1} (solubility); 32 ± 1 kg mol^{-1} (NMR)	Partial insertion of isoalloxazine region; ribityl chain limits depth	B	[26]
B2, riboflavin	βCD	Physical and microwave preparation; nanocomposite incorporation	1:1 from A_L profile	594.7 M^{-1}	NMR, FTIR, PXRD, DSC and microscopy; inclusion interpreted but direct spatial proof limited	B	[27]
B2 photoproduct, lumichrome	HPβCD	Phase-solubility complex, incorporated into collagen hydrogel	1:1	525 M^{-1}	A_L profile; increased solubility and photodynamic functionality	B	[33]
B2 photoproduct, lumichrome	HPγCD	Phase-solubility complex	1:1	77 M^{-1}	A_L profile; weaker solubilization than HPβCD	B	[33]
B5, DL-pantothenate hemicalcium	HPβCD	Aqueous 1:1 feed, electrospinning of concentrated solution	1:1 proposed	214.46 M^{-1}	A_L phase solubility, FTIR, PXRD, thermal analysis and docking; no 2D NMR	B	[41]
B6, pyridoxine	βCD	Kneading, co-precipitation and freeze-drying	Nominal 1:1	Not reported	FTIR, PXRD, DSC, ^1H NMR and UV–Vis; interaction/solid transformation, no 2D NMR	B	[43]
B6, unspecified vitamer	HPβCD	Competitive fluorescence displacement in aqueous solution	1:1 model	71 ± 4 L mol^{-1}	Indirect competition with fluorescent dyes; analytical use	C	[44]
B6, pyridoxal	βCD	Solution association used on Cu nanoclusters	1:1	3421.6 M^{-1}	Fluorescence fitting, FTIR and DFT; no NMR/ROESY	B	[46]
C, L-ascorbic acid	βCD	Kneading, co-precipitation and freeze-drying	Nominal 1:1	Not reported	FTIR, PXRD, DSC, ^1H NMR and UV–Vis; co-precipitation/freeze-drying most effective	B	[61]
C, L-ascorbic acid	βCD	High hydrostatic pressure, isolation of solid	Nominal 1:1 feed	Not reported	FTIR, UV–Vis and DSC; product loading ~50% of theoretical 1:1 maximum at ~300 MPa	C	[62]
